# A 15-year registry based follow up study of site specific cancer mortality among immigrants with type 2 diabetes in Sweden

**DOI:** 10.1038/s41598-026-39293-x

**Published:** 2026-02-13

**Authors:** Daniel N. Tollosa, Sol P. Juarez, Alessandra Grotta, Mikael Rostila

**Affiliations:** 1 Department of Public Health Sciences, Stockholm University, Stockholm, Sweden; 2 Centre for Health Equity Studies, Stockholm University/Karolinska Institutet, Stockholm, Sweden; 3 Aging Research Center, Karolinska Institutet/Stockholm University, Stockholm, Sweden

**Keywords:** Type 2 diabetes, Cancer mortality, Immigrant generations, Registry-based, Sweden, Cancer, Diseases, Endocrinology, Oncology, Risk factors

## Abstract

Cancer is a leading cause of death among individuals with type 2 diabetes (T2D), and immigrants in Europe face a higher T2D risk than native populations. We investigated mortality disparities in overall and eight specific T2D-related cancers among immigrants and native Swedes diagnosed with T2D between 2006 and 2021. An open cohort of 478,607 individuals aged ≥ 35 years at the time of T2D diagnosis (28% first-generation [G1] and 6% second-generation [G2] immigrants) was followed until December 31, 2023. Flexible parametric survival models were used to estimate hazard ratios (HRs), stratified by age at arrival and duration of residence for G1 immigrants. G1 immigrants, except those from Nordic countries, generally had lower overall cancer mortality than natives. However, mortality risks for specific cancers converged toward those of natives with longer residence in Sweden, and notably liver and endometrial cancer mortality were higher among those who arrived early in life compared with natives. In G2 immigrants, kidney and endometrial cancer mortality rates were elevated, particularly for those with Western (HR = 1.63) and Nordic (HR = 1.82) parental origins, respectively. Mortality rates from colorectal and liver cancers also appeared to increase among those more recently diagnosed with T2D. These findings underscore the need for strengthening integrated diabetes and cancer care and tailored support services for high-risk immigrant populations.

**Supplementary Information:**

The online version contains supplementary material available at 10.1038/s41598-026-39293-x.

## Introduction

Type 2 diabetes (T2D) is a chronic metabolic disorder that poses a significant global health burden^1^, resulting in increased incidence and mortality from several types of cancers, including liver, pancreatic, endometrial, breast, kidney, and bladder^2–4^. Recent studies in the U.S. and UK have shown that cancer is overtaking cardiovascular diseases (CVDs) as the primary cause of death among individuals with T2D^5–7^. The pathways behind the increased risk of cancer mortality in patients with T2D have not yet been established but are possibly due to a combination of interconnected factors, including medical (such as metabolic dysregulation and comorbidities), behavioral, lifestyle, and socioeconomic factors^8–10^.

Studies have revealed that the prevalence of T2D is greater among immigrants to Europe than among the native population, especially among those from South Asia and the Middle East^11^. Compared with host populations, they also tend to develop conditions at younger ages^12,13^. While Sweden’s universal healthcare system aims to provide equitable access to all residents, immigrants with chronic diseases, such as T2D, represent a potentially vulnerable population due to a multitude of structural constraints on disease management and care, including linguistic challenges, a lack of culturally sensitive care, and limited records of their health care history^14^. These challenges can lead to lower health service use and screening participation, as well as miscommunication of symptoms and delays in cancer diagnosis, which contribute further to disparities in cancer mortality. However, despite these challenges and the lower socioeconomic status of the immigrant population, which is often linked to poorer health and increased mortality risks, immigrants with T2D exhibit lower mortality rates, including for cancer, than their native counterparts do^12,15^.

In Sweden, the immigrant population constitutes approximately 20% of the total population and is characterized by considerable cultural, socioeconomic, and demographic diversity^16^. These diversities may lead to variations in cancer mortality risk factors and can be reflected in migrant-related factors, including generational factors, country of origin, age at arrival, and duration of residence dynamics. For example, immigrants who arrive at a young age have longer exposure to the receiving country’s environment and may have faster acculturation than those who migrate later in life. However, there is limited evidence on site-specific cancer mortality rates for distinct immigrant groups, including those with T2D. Bennet et al. recently reported a lower cancer mortality rate among immigrants with T2D in Sweden than among natives^12^; however, the results are presented only for all cancers combined. Given that T2D increases the risk of certain cancers than others and that cancer types are diverse in their etiologies and progressions, this lack of detailed analysis may obscure important disparities in cancer mortality between immigrant and native populations. This gap in knowledge is also particularly crucial to address, as healthcare for diabetic patients is increasingly moving toward integrated personalized care and treatment strategies^17^.

Therefore, this study aimed to examine whether immigrants diagnosed with T2D between 2006 and 2021 in Sweden exhibit different mortality risks for overall and eight specific T2D-associated cancers compared to native Swedes and whether these disparities are influenced by generational status, age at immigration, duration of residence, and calendar year of T2D diagnosis.

## Methods

### Data sources

Data were sourced from Swedish population-based registries. The Total Population Registry (TPR)^18^ provided data on participants’ country of birth, date of birth, sex, and migration. Socioeconomic factors (e.g., education, income, employment, and civil status) were obtained from the Longitudinal Database of Health, Insurance, and Labor Market Studies (LISA)^19^. The Multi-Generation Registry^20^ is used for extracting links of individuals with their biological parents. T2D cases were identified through the National Patient Registry (NPR)^21^, which contains International Classification of Diseases (ICD) coded diagnoses and administrative data (e.g., date of admission and discharge), and the Prescribed Drug Registry (PDR)^22^, which started in July 2005 and contains information about the patient, a product with its Anatomical Therapeutic Chemical (ATC) classification code, and prescription details (e.g., prescription and dispensing date). The Cause of Death Register^23^ provides death dates and ICD codes for underlying causes, which we accessed until December 31, 2023. All datasets were linked via pseudonymized personal identification numbers, which are assigned to all Swedish residents.

### Study population

Using an open cohort design, the study included all individuals diagnosed with T2D between 2006 and 2021. In line with previous studies^12,24^, the date for T2D diagnosis was either the date of first hospital discharge or outpatient contact for diabetes (*ICD-10*: E10-E15) in the NPR registry or their first glucose-lowering drug prescription (Anatomical Therapeutic Chemical (ATC) classification code starting with A10) in the PDR registry, whichever came first. Since T2D mostly occurs during mid-to-late adulthood, we excluded patients diagnosed with diabetes before the age of 35 to reduce the risk of including patients with type 1 diabetes. We also excluded immigrants who were diagnosed with T2D and died of cancer within two years after arrival at Sweden (*n* = 170), which aimed to reduce the likelihood of including those immigrants who arrived in Sweden nearly at the end-of-life stage with cancer. The selection of the study population and the exclusion criteria are presented in sFigure 2.

### Variables

The main outcomes of this study were mortalities for all-cancer combined (ICD-10: C00-C99) and eight site-specific cancers associated with T2D, including liver (ICD-10: C22), pancreas (ICD-10: C25), colorectal (ICD-10: C18-C21), esophageal (ICD-10: C15), kidney (ICD-10: C64), and bladder (ICD-10: C67) cancers, and sex-specific including breast (ICD-10: C50) and endometrial (ICD-10: C54) cancers in females.

Individuals were categorized into natives (those born in Sweden from parents who were also born in Sweden), the first immigrant generation (G1) (those born outside but immigrated to Sweden at a certain age), which were sub grouped by age at arrival (< 18, 18–35, and > = 35 years), the duration of residence in Sweden at the time of T2D diagnosis (< 15 and > = 15 years), and the second immigrant generation (G2) (those born in Sweden from at least one foreign-born parent). Additionally, immigrants were grouped into the following regions on the basis of their country of birth (for G1) or on parents’ birth country (for G2): Nordic (including Finland, Denmark, Norway and Iceland); Western (including all European countries excluding Nordic countries, North America, Australia and New Zealand); and non-Western (Latin America, the Middle East, Asia, and Africa) regions.

The covariates used for model adjustment included sex, education level (defined as the highest achievement level and classified into primary school, secondary school, collage/university, and unknown/missing), individual disposable income (divided into five quintiles), employment (employed and not employed or retired), and marital status (single, married, divorced, or widowed). All these variables were measured at the time of T2D diagnosis, and missing values were replaced by values from the previous calendar year. Comorbidities (yes/no), including cardiovascular diseases, obesity, and other disorders (pulmonary, renal or kidney, viral hepatitis, liver, depression and anxiety, and dementia), were considered if they occurred before the onset of T2D and may serve as a proxy for unmeasured clinical and pathological factors. These comorbidities were identified via ICD-10 codes from the patient registry (sTable 1). The estimates were also adjusted for the calendar period of T2D diagnosis.

### Statistics

Individuals (aged 35 years and above) were followed from the estimated date of diagnosis with T2D until they emigrated, died (with cancer or non-cancer), or reached the end of the study, i.e., on December 31, 2023, whichever occurred first. Survival time was calculated by subtracting the last follow-up date from the date of T2D diagnosis. Hazard ratios (HRs) and 95% confidence intervals (CIs) for all-combined and site-specific cancer mortalities were estimated for G1 – stratified by age at arrival and duration in Sweden – and G2 using flexible parametric models, with age at diagnosis as the time scale and three degrees of freedom for the baseline hazard. All models used natives as the reference group and were adjusted for individual disposable income, education status, marital status, employment status, comorbidities, and calendar period of T2D diagnosis (modeled as a restricted cubic spline). The models were fitted separately for each cancer site. We compared candidate models using the Akaike information criterion (AIC) and Bayesian information criterion (BIC), and the model with all predefined covariates consistently showed the best fit, and thus used in all final analyses. We also plotted the adjusted hazard ratios, estimated from the parametric models, for all-site and subsite cancer mortality, comparing immigrants (G1 and G2 separately) and natives as a function of the year of T2D diagnosis. Survival curve probabilities estimated from the fully adjusted model are also reported in the supplementary file. Two-sided p values < 0.05 were considered statistically significant.

While our conclusions are drawn mainly from the parametric models, owing to potential differences in baseline cancer mortality risk factors between immigrants and natives with T2D and the absence of information on important confounding factors, such as lifestyle variables, we conducted a complementary analysis by computing the age- and sex-standardized mortality ratio (SMR_atio_), which was calculated by dividing the cancer mortality rate among immigrants with T2D by the rate among immigrants without T2D and then dividing this ratio by the ratio of cancer mortality among natives with T2D to the rate among natives without T2D. Individuals without a history of diabetes (*n* = 2,803,666 (males); 2,823,691 (females)) were identified from the NPR and PDR registries, and similar follow-up criteria for age and calendar period as individuals with T2D were applied. The 2013 European Standard Population (ESP) was used to calculate age- and sex-standardized mortality rates.

$$\mathrm{SMR}_\mathrm{atio} =\:\frac{\mathrm{S}\mathrm{M}\mathrm{R}\:\mathrm{i}\mathrm{n}\:\mathrm{i}\mathrm{m}\mathrm{m}\mathrm{i}\mathrm{g}\mathrm{r}\mathrm{a}\mathrm{n}\mathrm{t}\mathrm{s}\:\mathrm{w}\mathrm{i}\mathrm{t}\mathrm{h}\:\mathrm{t}\mathrm{y}\mathrm{p}\mathrm{e}\:2\:\mathrm{d}\mathrm{i}\mathrm{a}\mathrm{b}\mathrm{e}\mathrm{t}\mathrm{e}\mathrm{s}\:/\:\mathrm{S}\mathrm{M}\mathrm{R}\:\mathrm{i}\mathrm{n}\:\mathrm{i}\mathrm{m}\mathrm{m}\mathrm{i}\mathrm{g}\mathrm{r}\mathrm{a}\mathrm{n}\mathrm{t}\mathrm{s}\:\mathrm{w}\mathrm{i}\mathrm{t}\mathrm{h}\mathrm{o}\mathrm{u}\mathrm{t}\:\mathrm{t}\mathrm{y}\mathrm{p}\mathrm{e}\:2\:\mathrm{d}\mathrm{i}\mathrm{a}\mathrm{b}\mathrm{e}\mathrm{t}\mathrm{e}\mathrm{s}\:\:}{\mathrm{S}\mathrm{M}\mathrm{R}\:\mathrm{i}\mathrm{n}\:\mathrm{n}\mathrm{a}\mathrm{t}\mathrm{i}\mathrm{v}\mathrm{e}\mathrm{s}\:\mathrm{w}\mathrm{i}\mathrm{t}\mathrm{h}\:\mathrm{t}\mathrm{y}\mathrm{p}\mathrm{e}\:2\:\mathrm{d}\mathrm{i}\mathrm{a}\mathrm{b}\mathrm{e}\mathrm{t}\mathrm{e}\mathrm{s}/\:\mathrm{S}\mathrm{M}\mathrm{R}\:\mathrm{i}\mathrm{n}\mathrm{n}\mathrm{a}\mathrm{t}\mathrm{i}\mathrm{v}\mathrm{e}\mathrm{s}\:\mathrm{w}\mathrm{i}\mathrm{t}\mathrm{h}\mathrm{o}\mathrm{u}\mathrm{t}\:\mathrm{t}\mathrm{y}\mathrm{p}\mathrm{e}\:2\:\mathrm{d}\mathrm{i}\mathrm{a}\mathrm{b}\mathrm{e}\mathrm{t}\mathrm{e}\mathrm{s}\:}$$

where SMR = Age- and sex-standardized cancer mortality rates.

### Sensitivity analyses

We also conducted sensitivity analyses by excluding individuals diagnosed with any cancer diagnosis found in the NPR register (ICD-9: 140–209; ICD-10: C00-C99) prior to their T2D diagnosis to evaluate the influence of preexisting cancer on our findings. This analysis revealed potential differences in the timing of cancer diagnosis relative to that of T2D between immigrant groups and native individuals and was performed for all-site combined and for colorectal, liver, pancreas, kidney, bladder, breast (females) and endometrial cancers. A total of 37,364 individuals were excluded, of whom 9,044 died of cancer.

Data analysis was performed via Stata software (Stata Ver.17, Stata Corp, College Station, Texas 77845 USA).

## Results

### Patient characteristics

The study included 478,607 individuals with T2D; 58% were males, and 28% and 6% were G1 and G2, respectively. There were 28,654 cancer deaths, with pancreatic cancer accounting for the highest proportion (18%) of the deaths. Compared with natives, immigrants in G1 and G2 were relatively 7–8 years younger at estimated T2D diagnosis (median ages: 59 and 58 vs. 66 years, respectively). Socioeconomic characteristics were comparable between natives and G2; however, a higher proportion of G1 had lower disposable income (lowest income quintile: 35% vs. 14%), were married (59% vs. 51%), and were unemployed (64% vs. 54%). The percentage of individuals with primary school (as their highest level of education) was higher in G1 (31.6%) than in natives (28.1%) and G2 (20.2%). Cardiovascular diseases were the most common comorbidity among both immigrants (G1 and G2) and natives (Table 1).

**Table 1:** Number of person-years, all-cancer deaths, and base-line characteristics of the study population by immigrant groups with type 2 diabetes, Sweden, 1990–2021.

Characteristics		Natives	G1: All	G1: Age at immigration (in years)			G1: Time in Sweden (in years)		G2
				< 18	18–35	>=35	< 15	>=15	
Study population	Frequency	313,899	135,787	17,094	62,998	53,245	46,824	86,683	28,921
Person-years	-	2,503,034	1,048,753	129,928	497,766	402,937	353,951	676,679	222,627
All-cancer deaths	Frequency	21,401	6,074	795	3,060	1,878	906	4,827	1.232
Age at T2D diagnosis (in years)	Median (IQR)	66 (57, 73)	59 (50, 69)	56 (46, 66)	59 (49, 70)	60 (51, 69)	51 (43, 61)	63 (54, 72)	58 (50, 66)
Sex (n, %)	Male	187,488 (59.7)	73,356 (54.0)	9,349 (54.7)	34,117 (54.2)	28,767 (54.1)	24,475 (52.5)	47,758 (55.1)	17,382 (60.1)
	Female	126,411 (40.3)	62,431 (46.0)	7,745 (45.3)	28,881 (45.8)	24,478 (45.9)	22,179 (47.5)	38,925 (44.9)	11,539 (39.9)
Disposable income (quintiles) (n, %)	Q1 (Lowest)	44,246 (14.1)	47,641 (35.1)	2,962 (17.3)	15,156 (24.0)	28,881 (54.3)	24,265 (52.1)	22,734 (26.2)	3,807 (13.1)
	Q2	63,076 (20.1)	27,937 (20.6)	3,378 (19.8)	14, 402 (22.9)	9,449 (17.8)	6,553 (14.1)	20,676 (23.9)	4,634 (16.1)
	Q3	68,250 (21.7)	22,170 (16.3)	3,186 (18.6)	12,160 (19.3)	6,271 (11.7)	6,171 (13.2)	15,446 (17.8)	5,260 (18.2)
	Q4	67,967 (21.6)	20,841 (15.3)	3,861 (22.6)	11,608 (18.4)	5,086 (9.5)	5,663 (12.1)	14,892 (17.2)	6,980 (24.1)
	Q5 (Highest)	70,360 (22.4)	17,184 (12.7)	3,707 (21.7)	9,672 (15.3)	3,554 (6.7)	3,988 (8.5)	12,935 (14.9)	8,239 (28.5)
	Missing	-	14	-	-	14	14	-	1
Education status (n, %)	Primary school	88,089 (28.1)	42,966 (31.6)	4,715 (27.6)	20,840 (33.1)	16,502 (30.9))	13,343 (28.5)	28,714 (33.1)	5,842 (20.2)
	Secondary school	153,409 (48.9)	46,609 (34.3)	8,917 (52.2)	25,160 (39.9)	11,516 (21.6)	9,447 (20.2)	36,146 (41.7)	15,387 (53.2)
	College/University	71,508 (22.8)	33,961 (25.0)	3,352 (19.6)	15,885 (25.2)	14,227 (26.7)	13,743 (29.4)	19,721 (22.7)	7,550 (26.1)
	Unknown/Missing	893 (0.28)	12,237 (9.1)	110 (0.6)	1,113 (1.8)	10,986 (20.8)	10,107 (21.8)	2,102 (2.4)	142 (0.5)
Marital status (n, %)	Single	74,619 (23.8)	14,504 (10.7)	4,654 (27.2)	6,025 (9.6)	3,614 (6.8)	4,105 (8.8)	10,188 (11.7)	9,689 (33.5)
	Married	160,928 (51.3)	80,479 (59.3)	7,960 (46.6)	36,026 (57.2)	35,364 (66.4)	33,132 (70.9)	46,218 (53.3)	12,699 (43.9)
	Divorce	56,604 (18.0)	29,832 (21.9)	3,769 (22.0)	16,757 (26.6)	8,802 (16.5)	6,043 (12.9)	23,285 (26.9)	5,665 (9.6)
	Widowed	21,425 (6.8)	10,793 (8.0)	699 (4.1)	4,109 (6.5)	5,397 (10.2)	3,333 (7.2)	6,872 (7.9)	858 (3.0)
Employment status (n, %)	Unemployed or retired^a^	168,820 (53.7)	86,561 (63.8)	8,057 (47.1)	36,257 (57.5)	40,202 (75.6)	31,963 (68.6)	52,553 (60.6)	11,394 (39.4)
	Employed	143,209 (45.6)	49,054 (36.1)	9,024 (52.7)	26,642 (42.3)	12,998 (24.3)	14,675 (31.4)	33,989 (39.2)	17,469 (60.4)
	Missing	1,870	158	13	99	31	2	141	57
Calendar period (year) at type 2 diabetes (n, %)	2006–2009	60,989 (19.4)	25,565 (18.8)	2,781 (16.3)	12,625 (20.0)	9,292 (17.5)	7,177 (15.4)	17,521 (20.2)	4,649 (16.1)
	2010–2013	69,567 (22.2)	27,529 (20.3)	3,343 (19.6)	13,227 (21.0)	10,356 (19.4)	8,243 (17.7)	18,683 (21.6)	5,695 (19.7)
	2014–2017	79,910 (25.5)	35,335 (26.0)	4,304 (25.2)	15,457 (24.5)	15,080 (28.4)	13,490 (28.9)	21,351 (24.6)	7,341 (25.4)
	2018–2021	103,433 (32.9)	47,358 (34.8)	6,666 (39.0)	21,689 (34.4)	18,517 (34.7)	17,744 (38.0)	29,128 (33.6)	11,236 (38.8)
Comorbidity (n, %)	Cardiovascular diseases and obesity ^b^	108,583 (34.6)	35,984 (26.5)	4,684 (27.4)	17,291 (27.5)	12,877 (24.1)	7,937 (16.9)	26,915 (31.1)	7,965 (27.5)
	Others disorders ^c^	35,435 (11.3)	12,995 (9.6)	2,062 (12.1)	6,512 (10.3)	4,020 (7.5)	2,328 (5.0)	10,266 (11.8)	3,341 (11.6)

^a^ Unemployed and retired individuals at T2D diagnosis were grouped together to form a stable group for analysis, as both are not active in the labor market.

^b^ Cardiovascular-diseases and obesity were grouped together due to their strong interrelationship, as obesity is major risk factor for CVD. Individuals in this category could have either condition or both.

^c^ Include disorders of the pulmonary system, chronic kidney diseases, liver diseases, viral hepatitis, depression and anxiety, dementia.

### Adjusted HRs for all sites and sub-cancer types by immigrant background

**Fig. 1:**
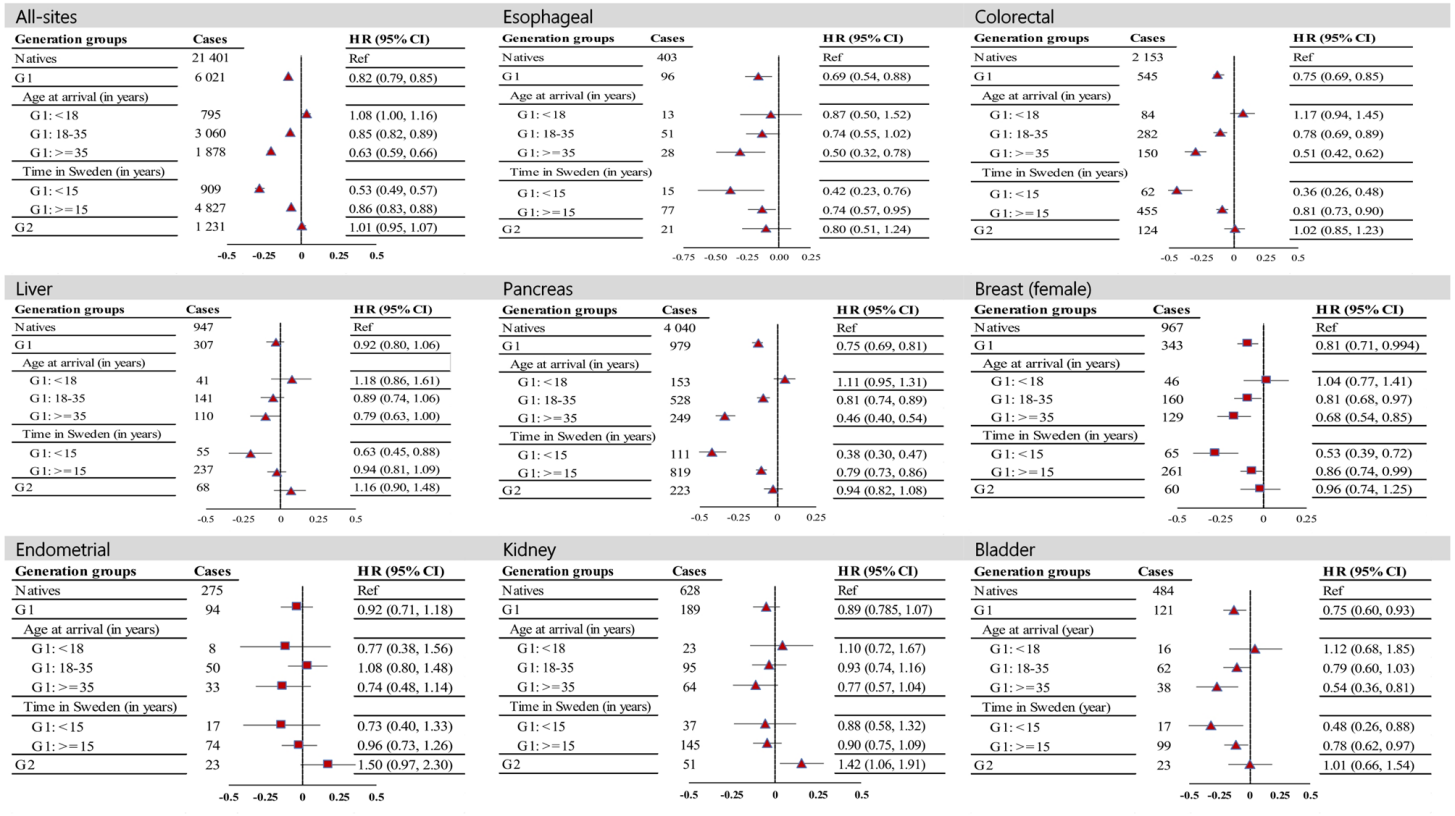
Hazard ratios (HRs) with 95% confidence intervals (CIs) for all-site and site-specific cancer mortality among immigrant groups with type 2 diabetes, diagnosed at age 35 years or older between 2006 and 2021, compared to native counterparts in Sweden. HRs are adjusted for age, calendar period, socioeconomic status (income, education, employment status), marital status, and selected comorbidities, including cardiovascular diseases (CVDs), obesity, and other conditions such as pulmonary, renal or kidney disorders, viral hepatitis, liver diseases, depression and anxiety, and dementia. Plots use a logarithmic scale: triangles represent both sexes combined, while rectangles indicate females only. Abbreviations: G1 (First generation), G2 (Second generation).

G1 immigrants generally exhibited either nonsignificant differences or lower HRs for all-cancer combined and for site-specific cancers. Among G2 immigrants, mortality rates did not differ significantly from those of native Swedes, with the exception of elevated mortality rates for kidney and endometrial cancer with Western (HR = 1.63; 95% CI: 1.02–2.66) and Nordic (HR = 1.82; 95% CI: 1.13–2.92) parental origins, respectively (Fig. 1; Table 2).

**Table 2:** Number of cases and HR (95% CI) for all-site cancers and site-specific cancer mortality in first-generation (G1) and second-generation (G2) immigrants with type 2 diabetes by country of origin, compared to native’s counterpart, Sweden, 2006–2021.

** Cancer type**	**Natives (Ref)**	**First-generation (G1)**						**Second-generation (G2)**					
		**Nordic**		**Western excl. Nordic**		**non-Western**		**Nordic**		**Western excl. Nordic**		**non-Western**	
	**Cases**	**Cases**	**HR (95% CI)**	**Cases**	**HR (95% CI)**	**Cases**	**HR (95% CI)**	**Cases**	**HR (95% CI)**	**Cases**	**HR (95% CI)**	**Cases**	**HR (95% CI)**
All site	21 401	2,215	1.01 (0.97, 1.06)	2 166	**0.92 (0.88, 0.97) **	1 693	**0.54 (0.52, 0.58) **	805	0.97 (0.91, 1.04)	379	1.02 (0.92, 1.13)	18	0.96 (0.60, 1.52)
Esophageal	403	43	1.11 (0.79, 1.53)	24	**0.57 (0.38, 0.88) **	30	**0.45 (0.30, 0.68) **	13	0.70 (0.40, 1.22)	7	0.85 (0.40, 1.79)	-	NR
Colorectal	2 153	200	0.92 (0.79, 1.07)	206	0.88 (0.75, 1.02)	145	**0.48 (0.40, 0.57) **	77	0.94 (0.75, 1.18)	43	1.15 (0.85, 1.56)	-	NR
Liver	947	100	1.07 (0.87, 1.32)	93	0.93 (0.74, 1.15)	115	**0.79 (0.64, 0.99) **	46	1.11 (0.82, 1.49)	20	1.10 (0.71, 1.72)	-	NR
Pancreas	4 040	406	1.04 (0.94, 1.16)	352	**0.83 (0.74, 0.93) **	229	**0.41 (0.36, 0.48) **	161	0.99 (0.85, 1.17)	57	0.77 (0.59, 1.00)	-	NR
Breast: females	967	112	0.98 (0.80, 1.20)	113	0.91 (0.74, 1.11)	113	**0.60 (0.47, 0.75) **	34	0.80 (0.57, 1.14)	24	1.30 (0.86, 1.95)	-	NR
Endometrial	275	33	1.02 (0.71, 1.48)	34	1.03 (0.71, 1.49)	27	0.65 (0.42, 1.01)	19	**1.82 (1.13, 2.92) **	-	NR	-	NR
Kidney	628	75	1.19 (0.92, 1.52)	74	1.09 (0.85, 1.40)	42	**0.46 (0.33, 0.65) **	32	1.32 (0.92, 1.91)	17	**1.63 (1.02, 2.66) **	-	NR
Bladder	484	43	0.78 (0.57, 1.07)	51	0.92 (0.68, 1.23)	28	**0.51 (0.33, 0.77) **	12	0.74 (0.42, 1.31)	9	1.37 (0.71, 2.67)	-	NR

HRs were adjusted age, calendar period, SES (income, education, employment status), marital status, and selected comorbidities (CVDs, obesity, and other disorders including pulmonary, renal or kidney, viral hepatitis, liver, depression and anxiety, and dementia).

Significant results (*p* < 0.05) highlighted in bold.

Nordic (Finland, Denmark, Norway, Iceland), Western excl. Nordic (all Europe excl. Nordic, USA, Canada, and Oceanian including Australia and New Zealand), and non-Western (Latin America, Asia, Africa and the Middle East).

Not reported (NR) **-** The number of cases were too small (*n* < 5).

Subgroup analyses within G1 revealed that HRs were elevated for all-site, colorectal, liver, pancreas, kidney, and bladder cancers who arrived before age 18 than for native population, although the differences were not statistically significant. Among those who arrived after the age of 18, the HRs were generally lower, or the differences were not statistically significant (Fig. 1). By the time in Sweden at T2D diagnosis, mortality among immigrants tends to converge toward that of natives with a longer duration of residence in Sweden. This pattern is consistent for the majority of subsite cancer types – HRs either shifted from significantly lower levels to nonsignificant differences (e.g., in the case of the liver) or remained lower but moved closer to the rate in natives. The most significant shifts were observed in colorectal and pancreas cancers, where the mortality rates increased by approximately 35–40% in those who had lived in Sweden for 15 years or more at the time of T2D diagnosis compared with those who had lived for less than 15 years (Fig. 1).

**Fig. 2:**
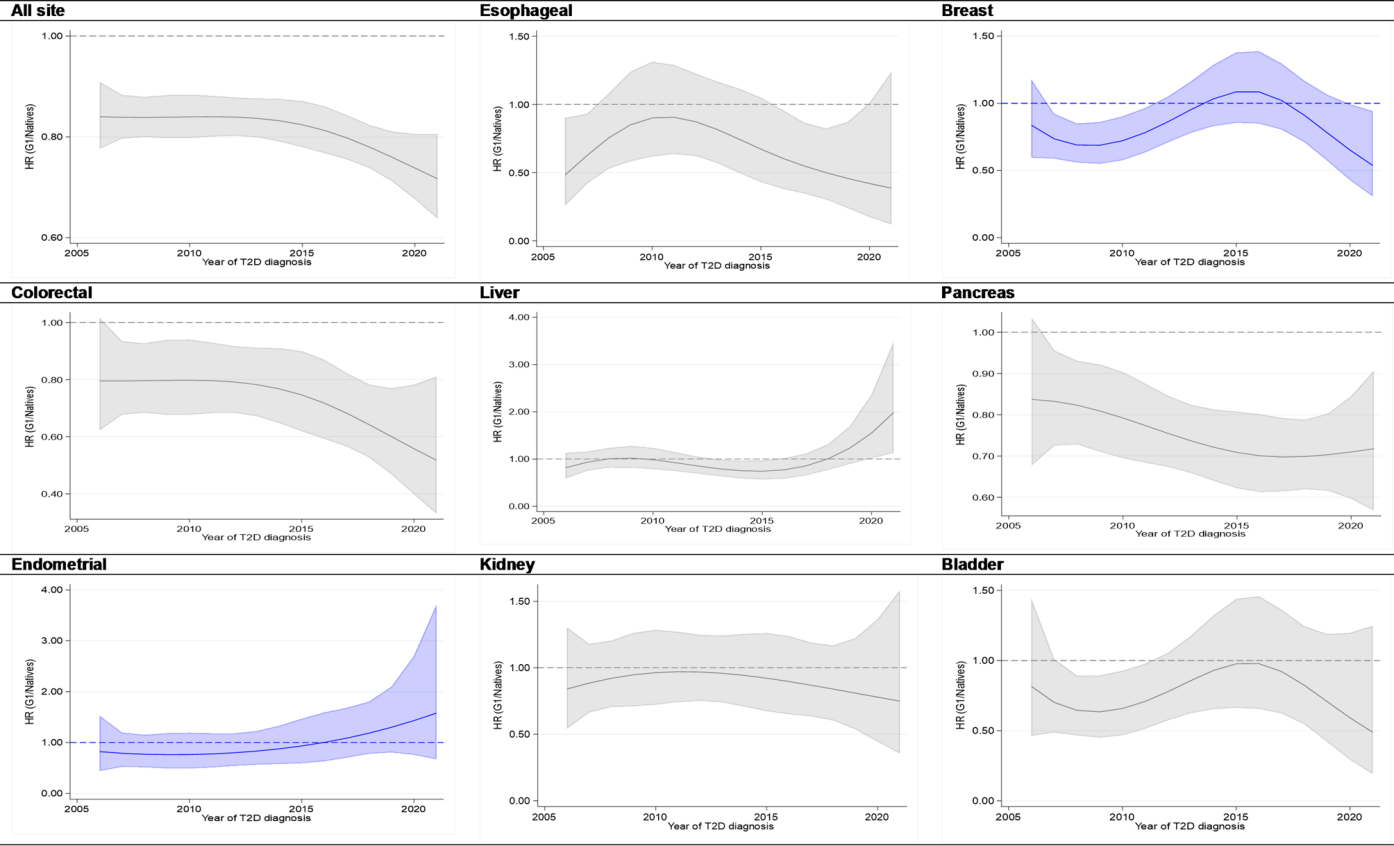
Adjusted hazard ratios for overall and site-specific cancer mortality among patients with type 2 diabetes aged 35 years and older, comparing first-generation immigrants (G1) to native Swedes based on the year of type 2 diabetes diagnosis between 2006 and 2021. Color: Gray (both sexes combined) and Blue (females only). Adjustments were made for age, calendar period, socioeconomic status (income, education, employment status), marital status, and selected comorbidities, including cardiovascular diseases (CVDs), obesity, and other disorders (pulmonary, renal or kidney disorders, viral hepatitis, liver diseases, depression and anxiety, and dementia).

**Fig. 3:**
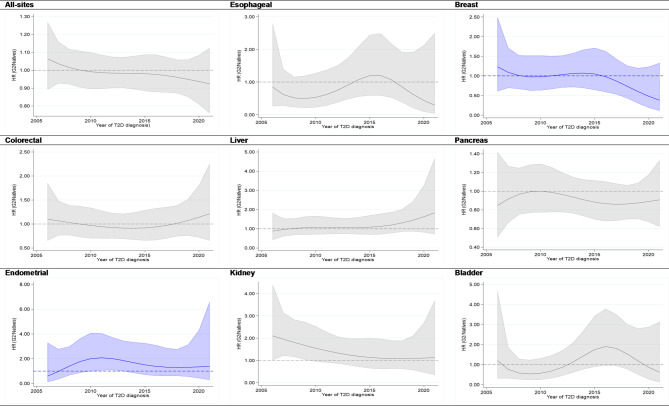
Adjusted hazard ratios for overall and site-specific cancer mortality among patients with type 2 diabetes aged 35 years and older, comparing second-generation immigrants (G2) to native Swedes based on the year of type 2 diabetes diagnosis between 2006 and 2021. Color: Gray (both sexes combined) and Blue (females only). Adjustments were made for age, calendar period, socioeconomic status (income, education, employment status), marital status, and selected comorbidities, including cardiovascular diseases (CVDs), obesity, and other disorders (pulmonary, renal or kidney disorders, viral hepatitis, liver diseases, depression and anxiety, and dementia).

Across the calendar years of T2D diagnosis, i.e., from 2006 to 2021, the adjusted hazard ratios (HRs) for all-site cancer mortality, comparing G1 to native individuals, remained relatively consistent, with an approximately 15% lower risk until 2015. It further declined and reached an approximately 25% lower risk in 2021. A similar pattern was observed for most subsite cancer types in G1, especially a declining trend after 2015. However, for certain cancers, such as liver and endometrial cancers in G1 and colorectal and liver cancers in G2, the mortality risk of these cancers appears to be greater in recently diagnosed individuals with T2D than in native individuals, although these differences have not yet reached statistical significance (Figs. 2 and 3).

### Supplementary analyses

Consistent with the findings of our primary analyses, the SMRs from the supplementary analyses suggested similar disparities in mortality rates among immigrant groups compared with those of natives. However, an exception was noted with elevated breast cancer mortality in G1 individuals from the Nordic region (SMR = 1.32, 95% CI: 1.10, 1.66) compared with that in natives, despite the primary analyses suggesting a nonsignificant difference for this group. The results of these supplementary analyses are presented in the supplementary files (sTable 2).

### Sensitivity analyses

Excluding individuals who were diagnosed with cancer prior to having T2D did not alter the conclusions of the main analysis. These results are presented in the supplementary files (sTable 3).

## Discussion

In this study of 478,607 individuals with T2D, we examined mortality disparities for overall and eight cancer types, possibly linked to T2D, between immigrant generations and natives in Sweden—subgroups defined further by migrant-related factors. Overall, with some exceptions, risks were lower or not significantly different for most cancers in both G1 and G2; however, subgroup analyses revealed elevated mortality in G1, particularly those who arrived before the age of 18 years and those who migrated from Nordic or other Western countries, for all sites and most subtypes studied. Additionally, mortality rates in G1 were converging toward those of natives the longer they lived in Sweden at the time of T2D diagnosis. An increasing pattern in mortality risk was also observed among immigrants recently diagnosed with T2D, particularly in the liver and endometrial in G1, and colorectal and liver in G2. These mortality disparities are likely associated with several interconnected pre- and postmigration factors, as well as potential differences in the management of T2D complications, treatment choices, and the impact of T2D itself on cancer progression. The observed difference in cancer mortality may also reflect variations in cancer incidence and survival rates between native and immigrant populations with T2D, influenced by disparities in lifestyle, environmental, and health care access and utilization.

The mortality advantage of G1 for various chronic diseases, including cancer, has been reported in previous studies^25^, but this advantage is paradoxical given their lower socioeconomic status, which is typically associated with increased mortality^26^. Likewise, we observed that immigrants with T2D, notably non-Western G1, had lower or nonsignificant differences in cancer mortality rates compared to native counterparts. Several factors may contribute to this observation, including the phenomenon of the “healthy migrant effect” which refers to self-selection in origin whereby younger and healthier individuals are more likely to emigrate, although some studies have reported that the mental and physical health conditions of immigrants in Sweden are not better than those of native individuals^27,28^.

Specifically, the lower mortality rate for liver cancer is an interesting observation in this study, given that the mortality rate for this cancer is often reported at higher rates among non-Western immigrants^29^. The higher prevalence of hepatitis B and hepatitis C virus infections, which are known risk factors for liver cancer, among non-Western immigrants in Sweden is well noted^30^. Thus, they may benefit from early detection and treatment of liver abnormalities during healthcare visits for diabetic management compared with the native population, potentially preventing progression to more severe conditions such as cirrhosis and hepatocellular carcinoma (HCC) and thus mortality. However, further investigation is warranted, as we did not find any empirical evidence supporting this hypothesis. Some medications used for T2D, such as metformin, are also associated with a reduced risk of liver cancer and improved survival rates^31^. The proportion of metformin (in combination with sulfonylureas) use is relatively greater than that of natives among immigrants in Sweden^12^, which may contribute further to the lower mortality rate of liver cancer among non-Western immigrants with T2D.

The subgroup analyses within G1 suggest that HRs converge toward those of natives with longer times in Sweden at T2D diagnosis. Similar trends have been observed for all-site cancer mortality in previous studies among immigrants diagnosed with T2D^12^. The influence of lifestyle changes and acculturation to Western culture—often described as high fat and processed food intake and a high rate of obesity—and improved access to cancer screening and health care likely contribute to the convergence of cancer mortality rates among immigrants toward the native population. Our findings of elevated cancer mortality rates among early-age migrants (i.e., before the age of 18) also align with previous studies in the general immigrant population^32^. Several factors may contribute to the increased risk of cancer mortality in this immigrant group. For example, relative to adults, young immigrants may face unique challenges and psychosocial stressors, including identity conflicts, acculturative stress, and potential discrimination^33–35^. All of these factors are linked to depression and anxiety, which affect their academic achievements at school and lead to low wages and hazardous jobs later in life. These factors, in turn, may contribute to cancer development and progression, especially when coupled with a diagnosis of other chronic diseases. The impact of depression and anxiety status on cancer incidence and mortality has been well documented^36^. Additionally, the fact that early-life migrants have greater cumulative exposure to potential environmental risk factors in receiving countries augments the risk of cancer over time. Epigenetic modifications following the new experience of the Western environment and lifestyle changes can have a profound influence on determining disease susceptibility later in life, including increasing cancer risk and mortality^37^.

Across the calendar years of T2D diagnosis, except for some cancers, mortality rates were lower in G1 than in the natives, particularly after 2015. This decline may be attributed to the substantial increase in migration flow into Sweden since 2010, which recorded an all-time high in 2015/16. The influx of young adult immigrants, who generally have a lower risk of cancer and mortality from it, may have contributed to this decline. However, as these migrants age, the mortality risk may reverse again; therefore, follow-up of cancer mortality risk among first-generation immigrants is warranted.

We also note that HRs for kidney and endometrial cancers were elevated in G2, particularly for those with Western and Nordic parental origins, compared with their native counterparts. Although previous studies have shown lower mortality rates of these cancers in G2 compared with native individuals^32^, the strong association of T2D and its complications with the progression of kidney and endometrial cancers^38,39^, as well as other socioeconomic factors, lifestyles (e.g., obesity and smoking status), and cultural differences between G2 individuals and native Swedes, may contribute to the observed disparities. Wändell et al. reported an elevated risk of end-stage chronic kidney disease (CKD), a common complication of T2D and a precursor of kidney cancer (or renal-cell carcinoma), among G2s in Sweden with European parental origin^40^. Given that the risk of mortality for kidney and endometrial cancer was found to be lower in G1, acculturation over time with Western lifestyle habits, combined with a lower socioeconomic profile than that of natives^41^, may have contributed to the loss of mortality advantage in G2, with certain cancers, such as kidney and endometrial cancers, being elevated. Studies also suggest that individuals born with low birth weights may have a greater risk of developing albuminuria and CKD later in life^42^, especially if they develop T2D. An increased risk of LBW across immigrant generations is evident in Sweden^43^.

While this study provides valuable insights into disparities in cancer mortality between individuals with foreign backgrounds and native Swedes diagnosed with T2D, it is important to note certain limitations, which underscore the need for further studies and careful interpretation of the findings. First, although major confounders and mediators were adjusted for when estimating cancer mortality disparities, other factors, including lifestyle (e.g., smoking), clinical and pathological characteristics (e.g., diabetic-related complications and medication use), certain infections such as hepatitis B and hepatitis C (particularly relevant for liver cancer mortality), access to culturally sensitive healthcare, and cancer screening participation, could modify cancer mortality risk among patients with type 2 diabetes. These factors were not available in our register data used for this study and thus could not be examined. Although adjustment for comorbidities, may serve as a proxy for some underlying health differences, it does not fully account for potential disparities in diabetes complications and infection-related risks between immigrants and natives, which may affect our estimates. Second, the definition of type 2 diabetes we applied has limitations in completely ascertaining T2D cases. For example, some individuals may have been prescribed antidiabetic medications for conditions other than T2D, such as metformin for polycystic ovarian syndrome, empagliflozin for heart failure, or GLP-1 receptor agonists for weight management. This may introduce misclassification bias. Additionally, our approach may under-capture individuals with diet-controlled T2D who are not receiving pharmacological treatment. While our definition is consistent with some previous studies that used similar registry datasets^12,24^, the case ascertainment has not been explicitly validated against the Swedish National Diabetes Register (NDR). Future research could benefit from linking prescription data with NDR records to improve classification accuracy. Third, for some of our findings, especially for cancers where the link between T2D and cancer progression is less clear and strongly linked to lifestyle factors such as smoking, our regression models may not fully explain mortality rate disparities; thus, further studies are recommended that account for additional background factors, including behavioral, lifestyle, infection history, and other clinical factors. Fourth, immigrant groups, especially those from non-Western regions, tend to be relatively young and small in size, which affects the number of cancer deaths reported in this study. This limitation may reduce the statistical power of our analyses, especially when examining subgroups defined by migrant-related factors. Therefore, although the proportion of older immigrants from non-Western regions has been rising in Sweden, where T2D-cancer morbidities and mortality rates are typically high, most of this group arrived in Sweden more recently than other immigrant groups (especially those from Nordic and other European countries); thus, they have not yet aged at comparable levels with the native population. This may affect our estimates, especially for G1 non-Western immigrants, although age differences were accounted for in the regression models. Moreover, while our study categorizes all migrants under 18 years as early-life migrants to ensure a stable statistical model, it is important to acknowledge that outcomes may vary within this group, e.g., those who migrate during early childhood versus teenage years, owing to potential differences in exposure type and duration to diabetes and cancer risk factors, as well as variations in the pace of acculturation in the host country. Fifth, over-coverage (where individuals are registered but not residing in the country) and salmon bias (where migrants return to their country of origin when they become ill or elderly) could introduce bias in our study. However, less than 1% of individuals have missing income data for three consecutive years during the follow-up period, which suggests a minimal effect on our conclusions. This low proportion may be due to immigrants with chronic health conditions being less likely to return to their home countries, where healthcare access and affordability are often limited. Finally, while we employed reasonable age criteria to identify only T2D patients in this study (those above 35 years at diagnosis), we cannot completely rule out the possibility of other types of diabetes, e.g., type 1 diabetes, as late-onset type 1 diabetes can also occur after the age of 35 years and may have been included in the analysis. However, given its relatively lower incidence rates in this age group^44^, it will not significantly impact our findings.

In conclusion, overall cancer mortality risk was generally lower in G1 individuals with T2D than in native Swedes; however, disparities existed across cancer subtypes and immigrant subgroups. Mortality risk converged to that of natives with longer durations in Sweden and was higher for most cancer subsites among those who arrived early in life. Compared with natives, G2 immigrants of Nordic and Western parental origin presented increased mortality risk for kidney and endometrial cancers, respectively. Strengthening integrated diabetes and cancer care and culturally tailored care services for high-risk immigrant groups are warranted.

## Supplementary Information

Below is the link to the electronic supplementary material.

Supplementary Material 1

## Data Availability

The datasets generated and/or analyzed during the current study are not publicly available owing to the sensitive nature of individual-level health data. However, the data are available from the corresponding author upon reasonable request and with permission from the responsible authority.

## Abbreviations

ATC: Anatomical therapeutic chemical
ESP: European Standard Population
LISA: Swedish version of the Longitudinal Database of Health, Insurance, and Labor: Market Studies
NPR: The National Patients Registry
PDR: Prescribed Drug Registry
SMR: Standardized mortality ratio
TPR: Total Population Registry
