# Supplementary material for: A 15-year registry based follow up study of site specific cancer mortality among immigrants with type 2 diabetes in Sweden
Source: Sci Rep. 2026 Feb 13;16:6493. doi: 10.1038/s41598-026-39293-x (PMC12910089; doi:10.1038/s41598-026-39293-x)
Supplement: Supplementary file 1 — Supplementary Material 1 [file 41598_2026_39293_MOESM1_ESM.docx]

**Supplementary files**

**A 15-year registry based follow up study of site specific cancer mortality among immigrants with type 2 diabetes in Sweden**

**sTable 1**: ICD-9 and ICD-10 codes for selected chronic diseases used as comorbidities in individuals diagnosed with type 2 diabetes, between 2006 and 2021, Sweden

| **Chronic health conditions** | **ICD-9 codes** | **ICD-10 codes** |
| --- | --- | --- |
| Obesity | 278 | E66 |
| Cardiovascular diseases (CVDs) | 390–459 | I10–I99 |
| Viral hepatitis and Liver diseases | 070, 571, 572, 573, 456A, 456B, 456C | B15, B16, B17, B18, B19  K70, K71, K72, K73, K74, K75, K76 |
| Renal or kidney diseases | 403A, 403B, 403X, 588A, V42A, V45B  582, 583, 585, 586, V56 | I120, I131, N032, N033, N034, N035, N036, N037, N052, N053, N054, N055, N056, N057, N11, N18, N19, N250, Z490, Z491, Z492, Z940, Z992  Q611, Q612, Q613, Q614 |
| Chronic pulmonary disease | 490, 491, 492, 493, 494, 495, 496, 500-508, 516, 517 | I278, I279, J40, J41, J42, J43, J44, J45, J46, J47, J60, J61, J62, J63, J64, J65, J66, J67, J68, J69, J701, J703 |
| Depression and Anxiety disorders | 296, 300 | F41, F32 |
| Dementia | 290, 331 | F02, F03, G30, G31 |

Abbreviations - ICD: International Classification of Diseases

The ICD codes were selected based on a review of literature and those used in the Charlson Comorbidity Index (CCI), which is widely used in epidemiological studies. Multiple codes for similar diseases were used to capture all relevant cases and minimize misclassification

| **All sites** | | | |
| --- | --- | --- | --- |
| 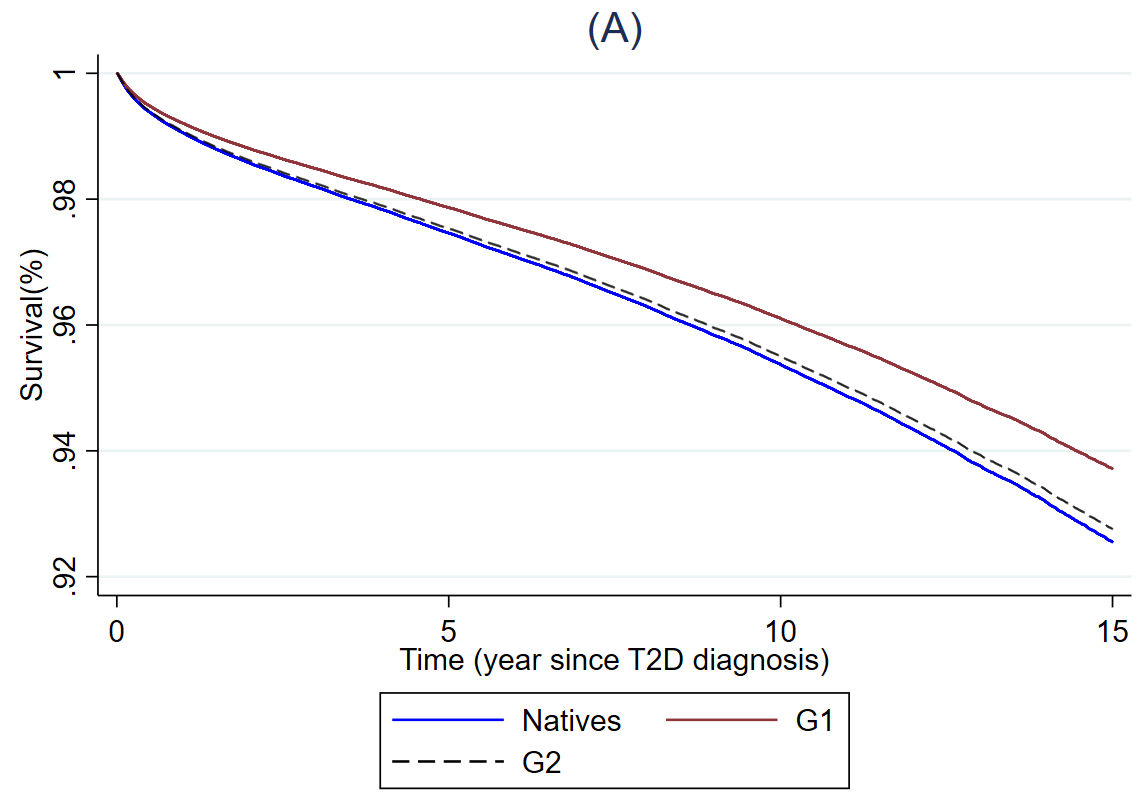 | 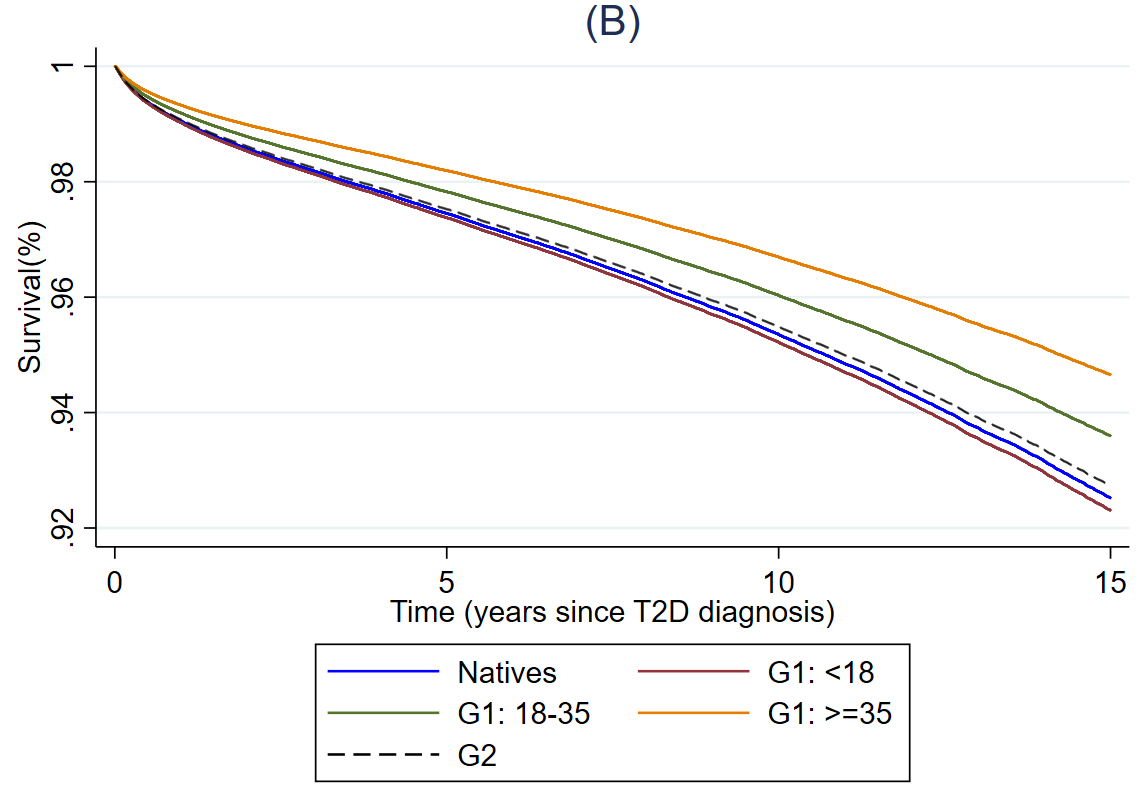 | | 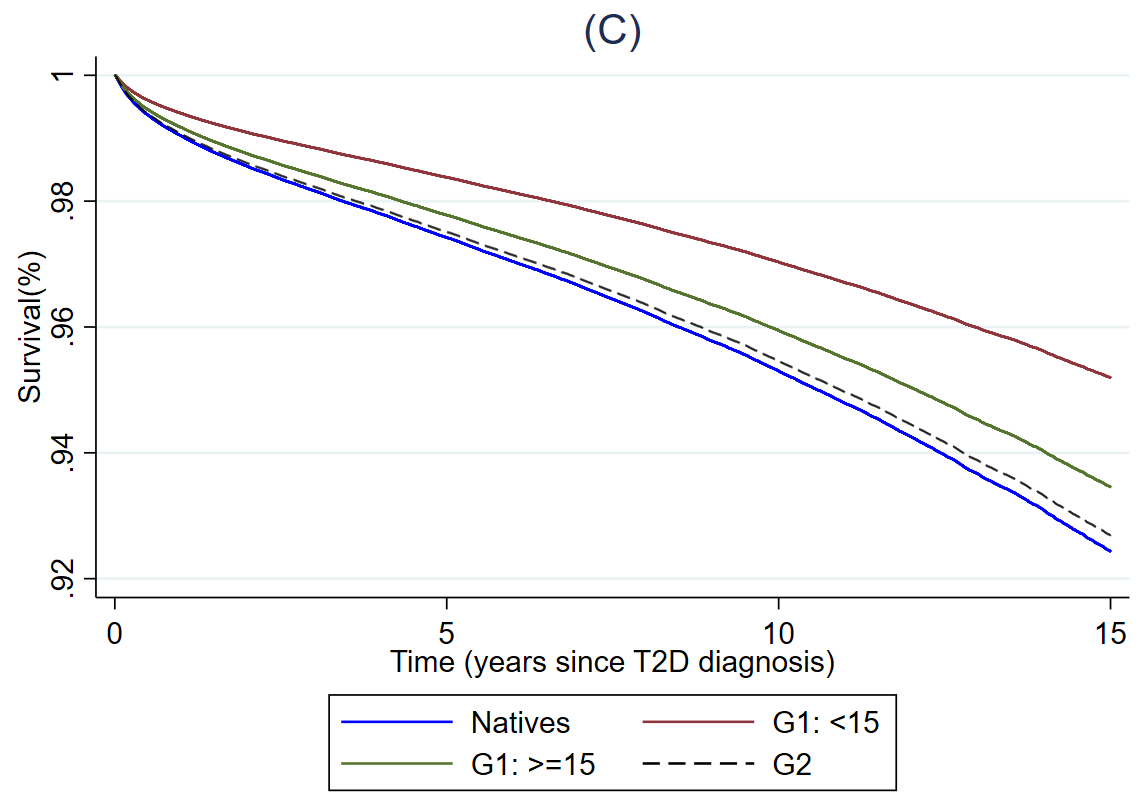 |
| **Esophageal** | | | |
| 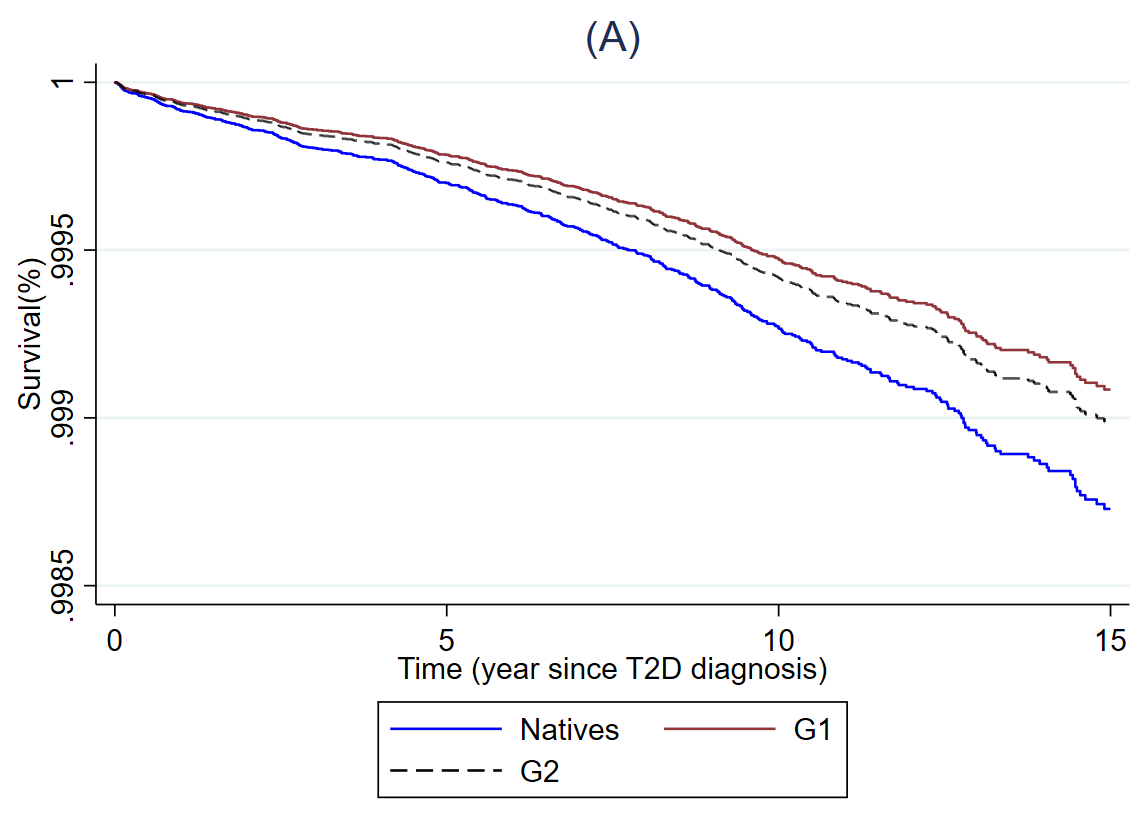 | | 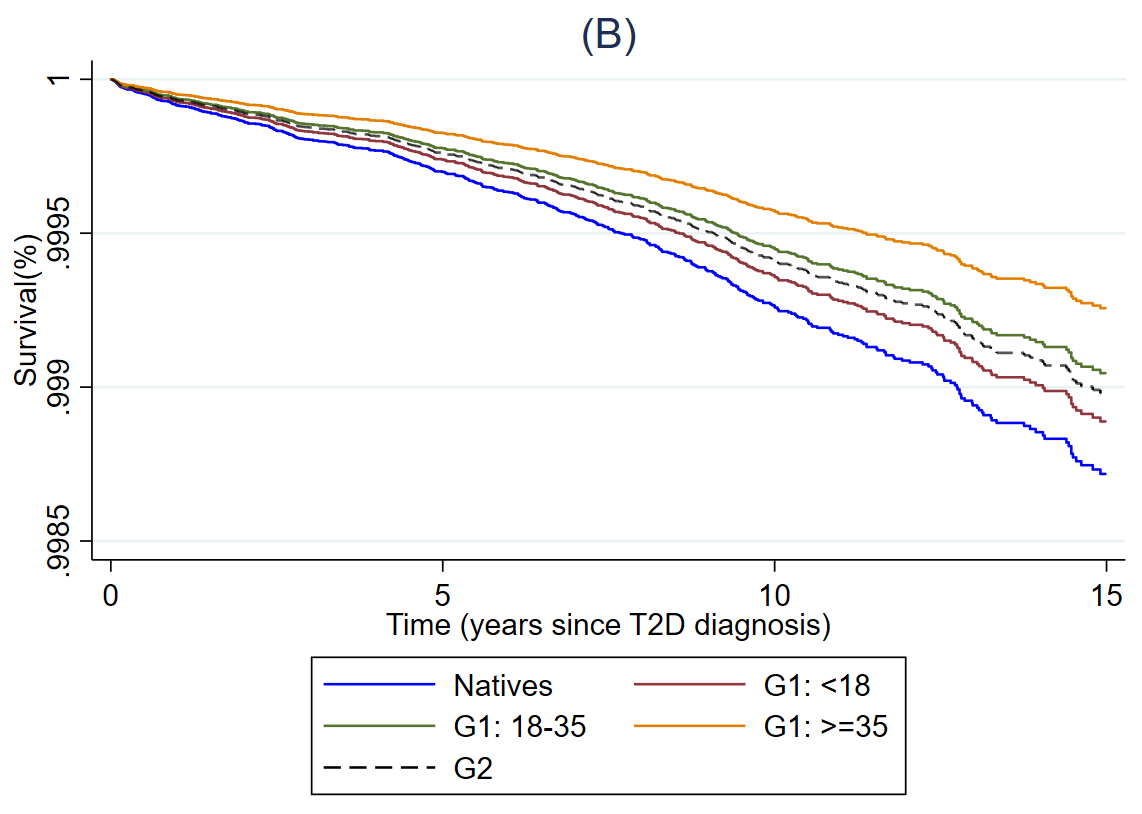 | 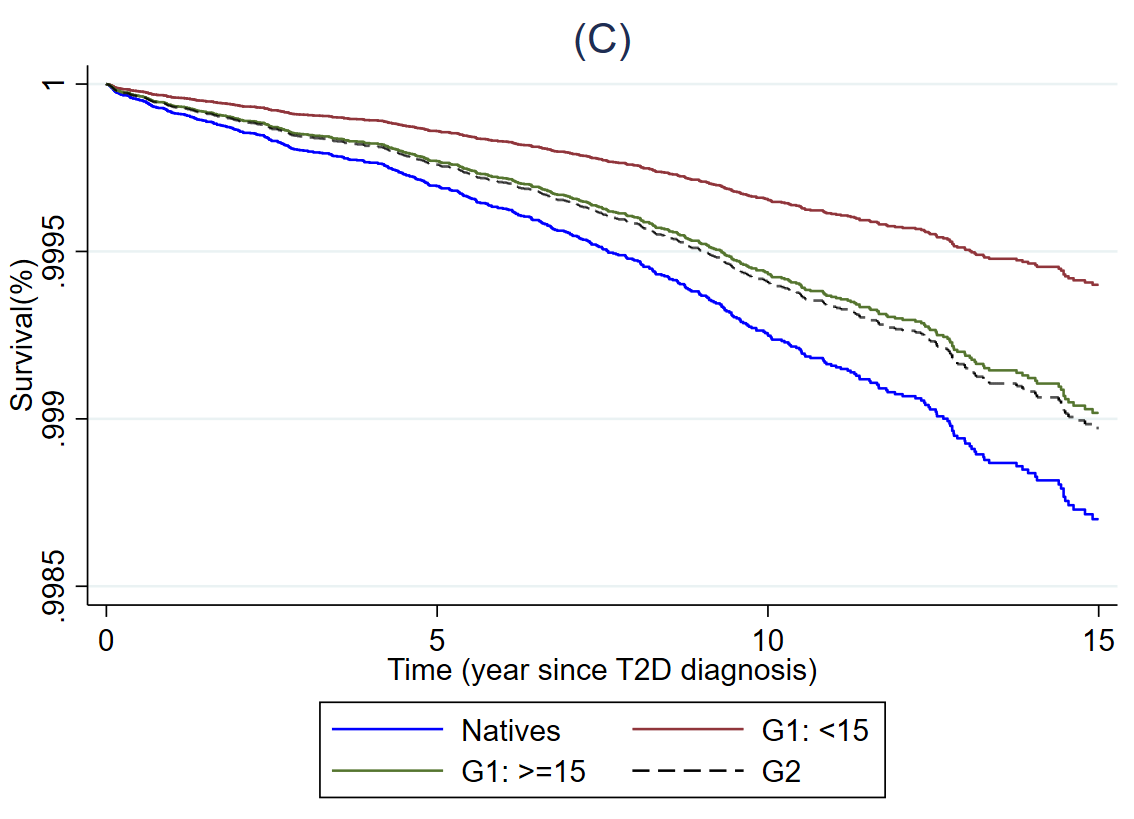 |
| **Colorectal** | | | |
| 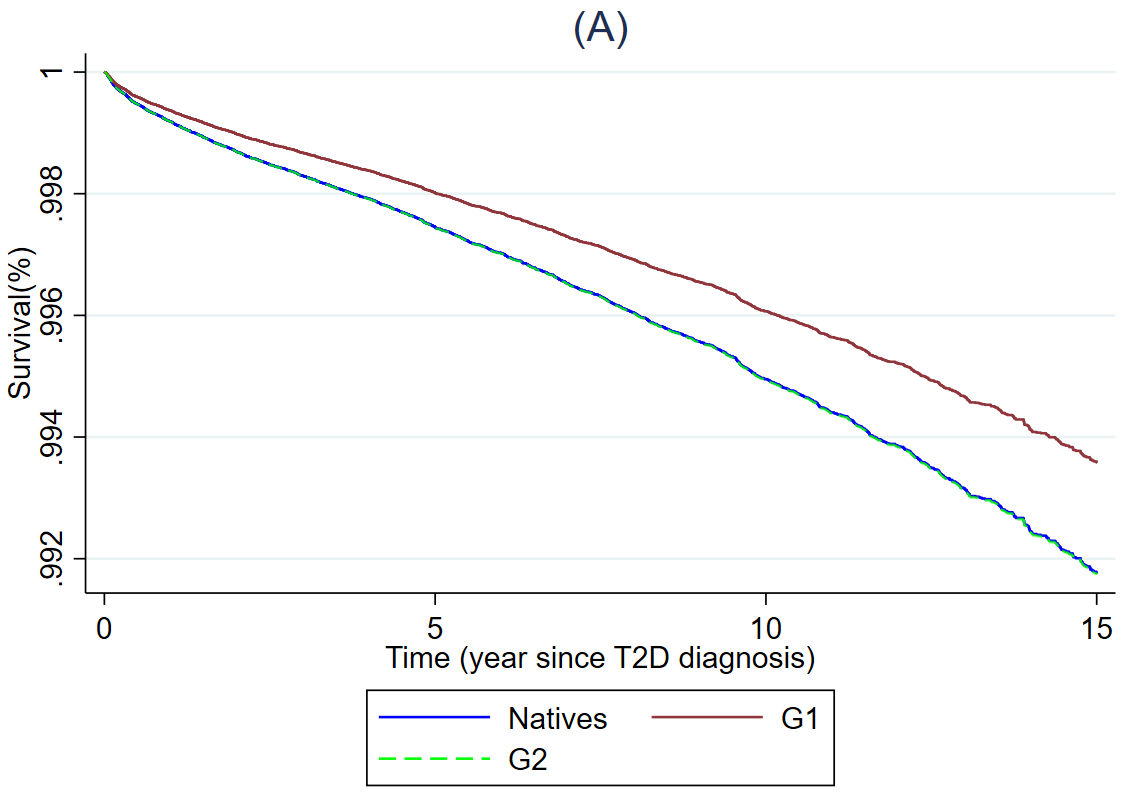 | | 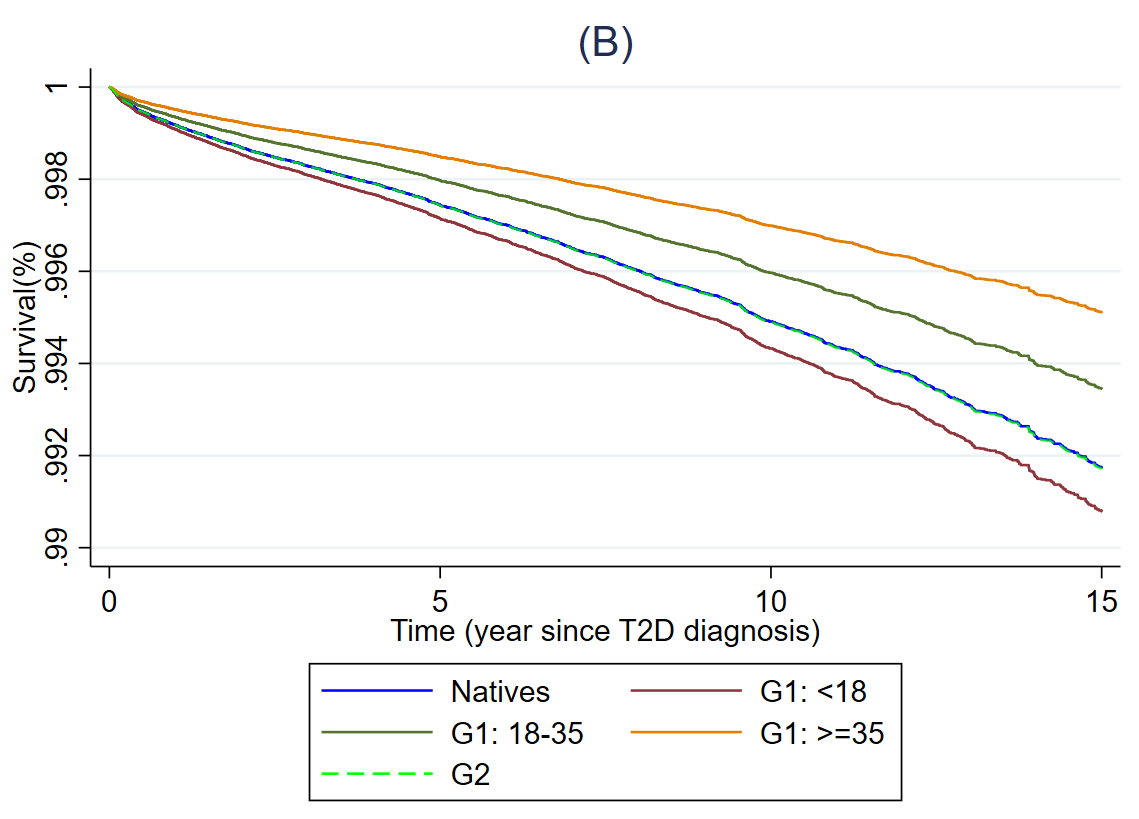 | 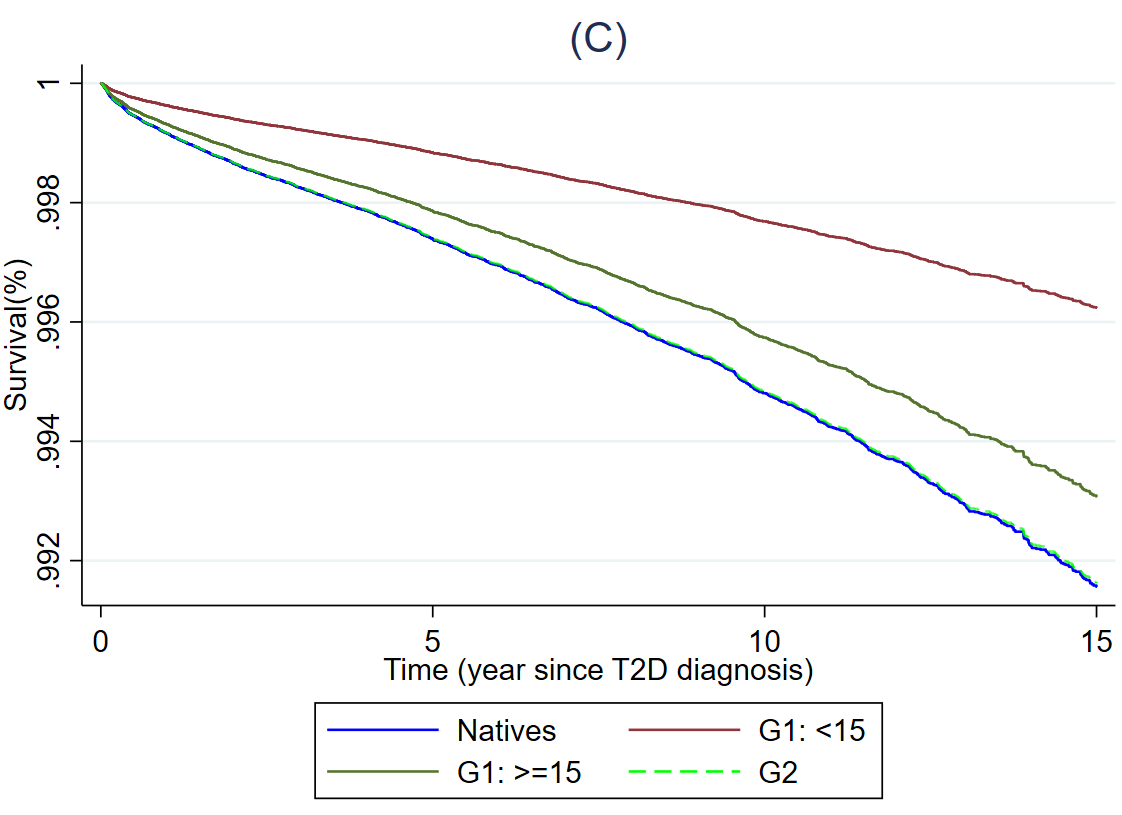 |
| **Liver** | | | |
| 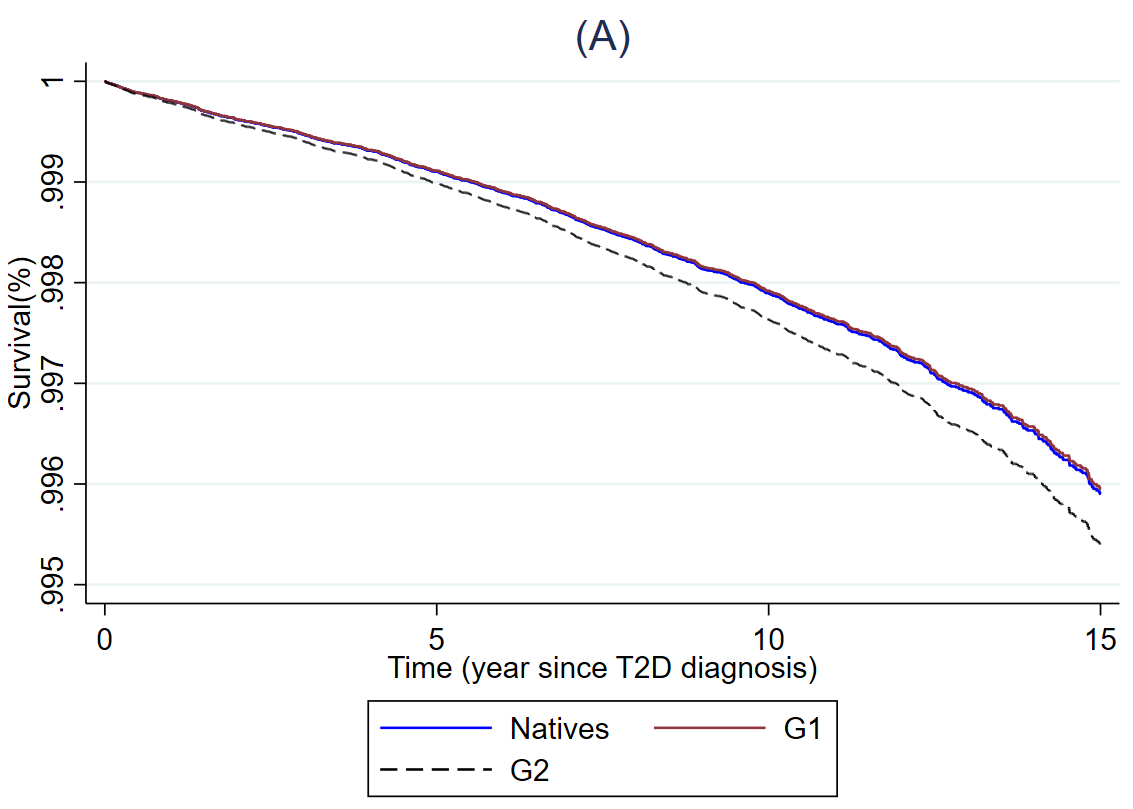 | | 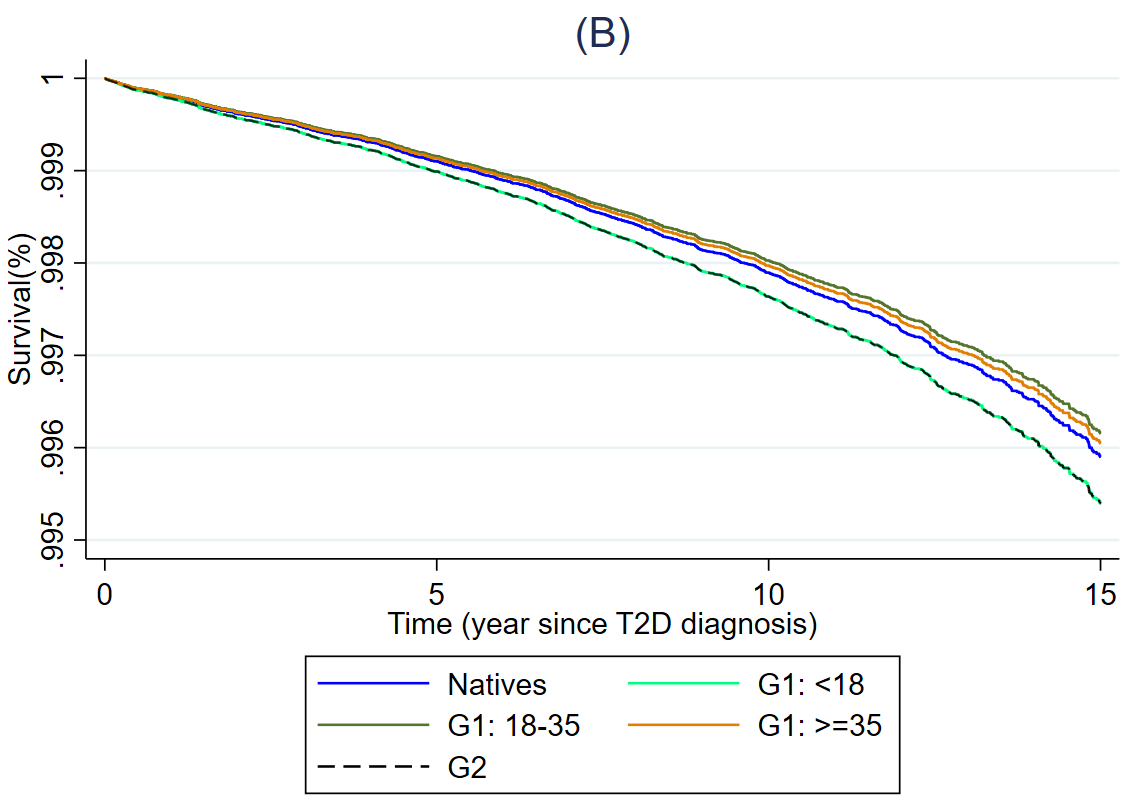 | 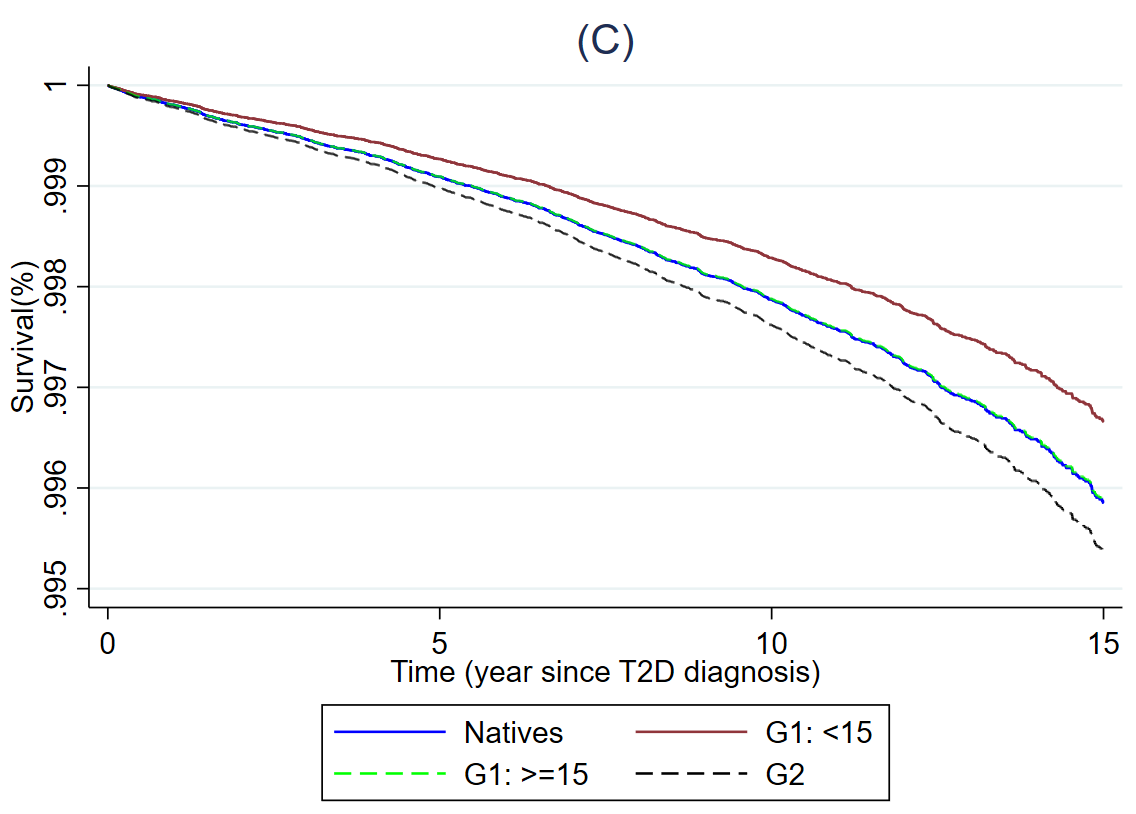 |
| **Pancreas** | | | |
| 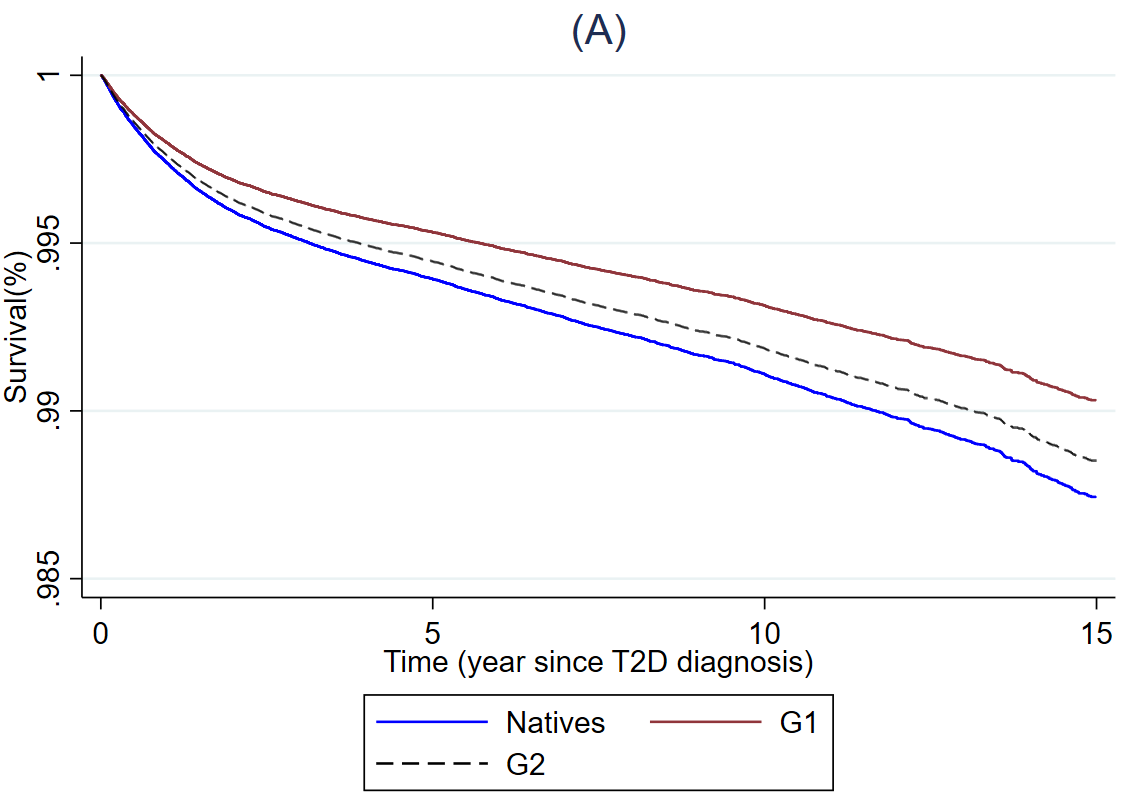 | | 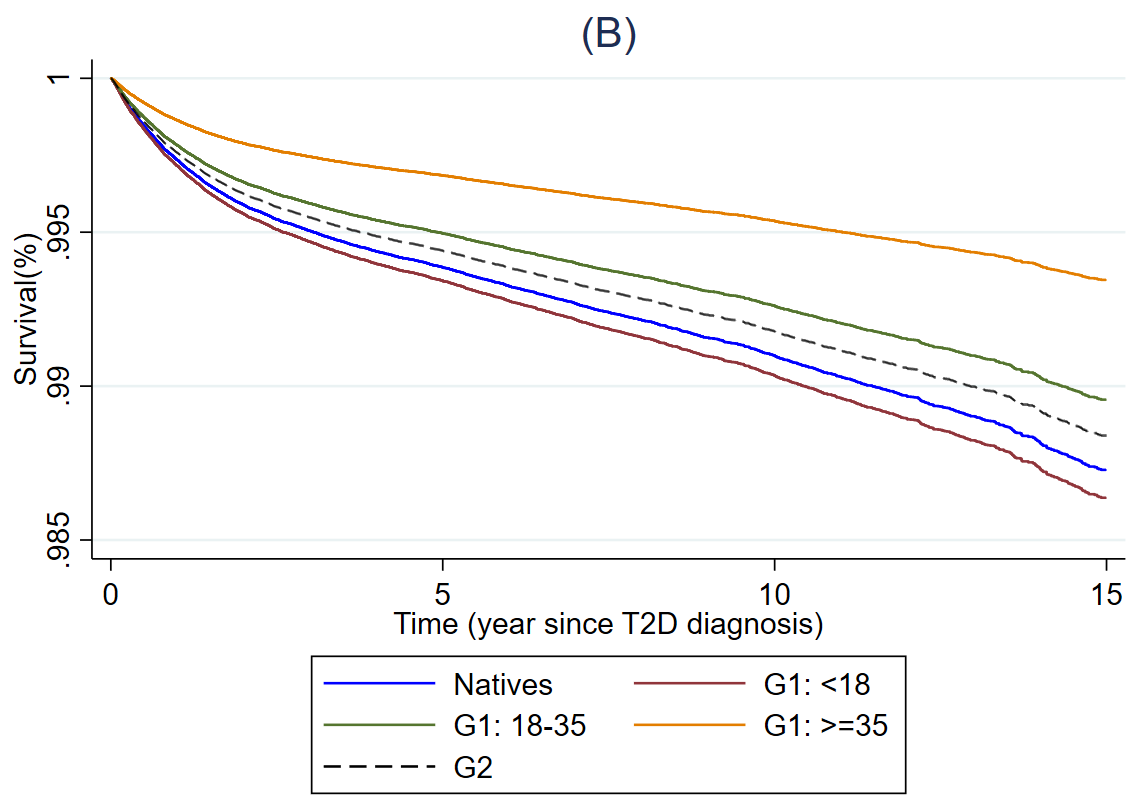 | 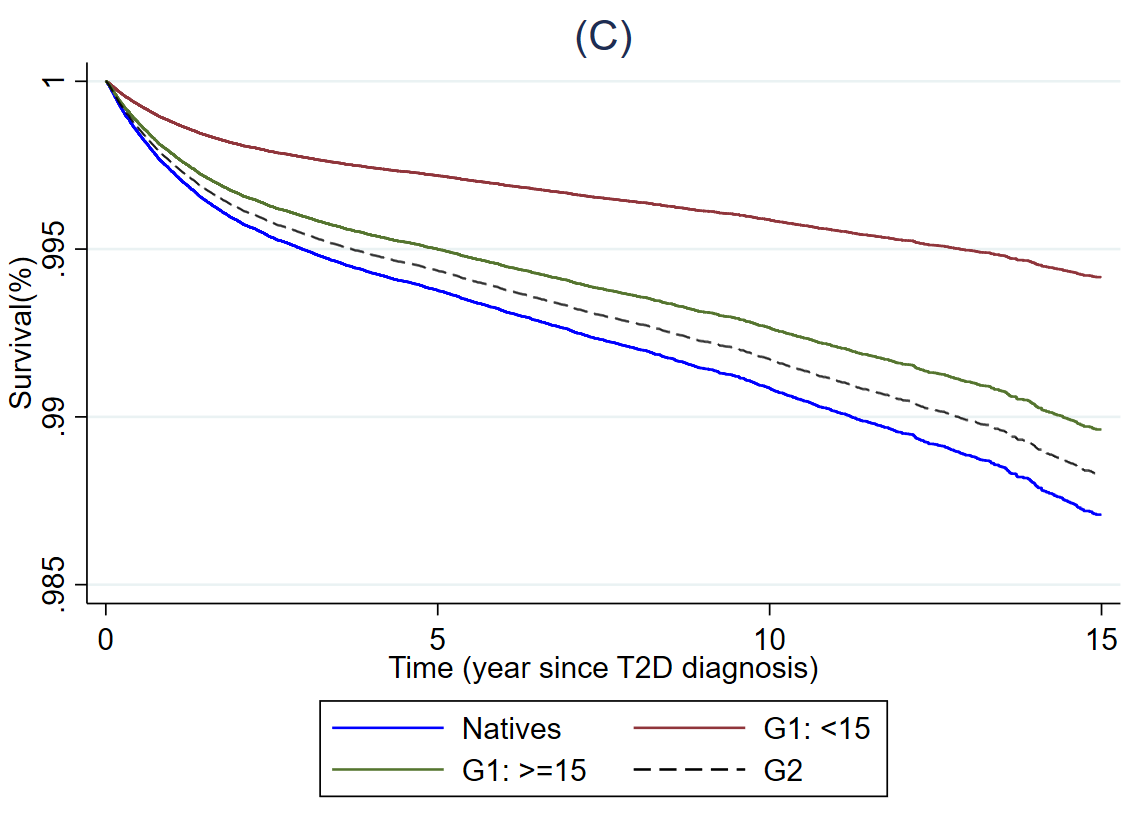 |
| **Kidney** | | | |
| 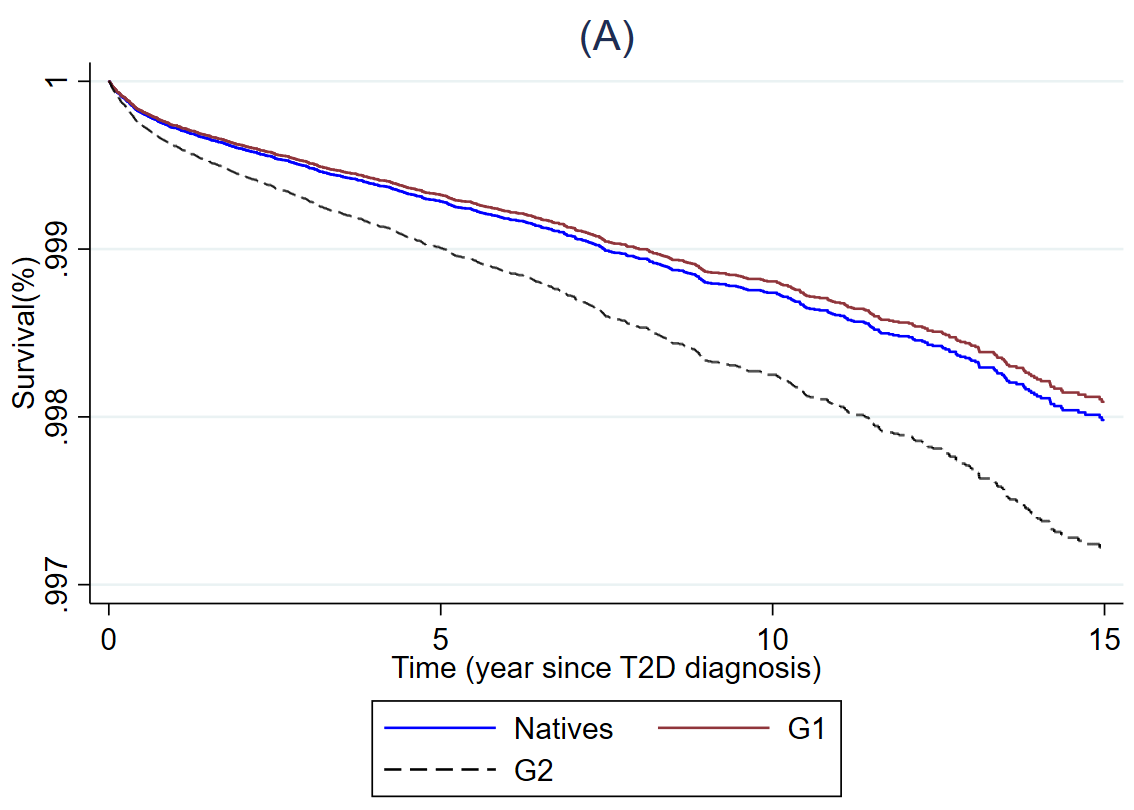 | | 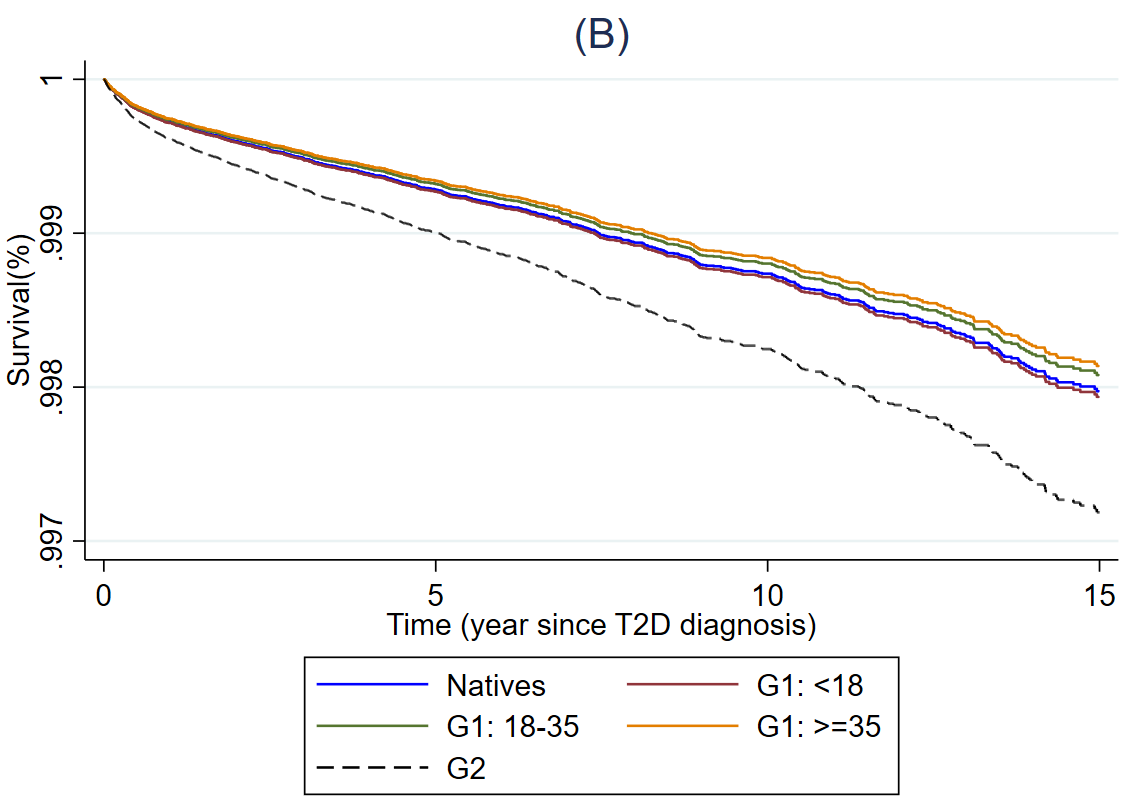 | 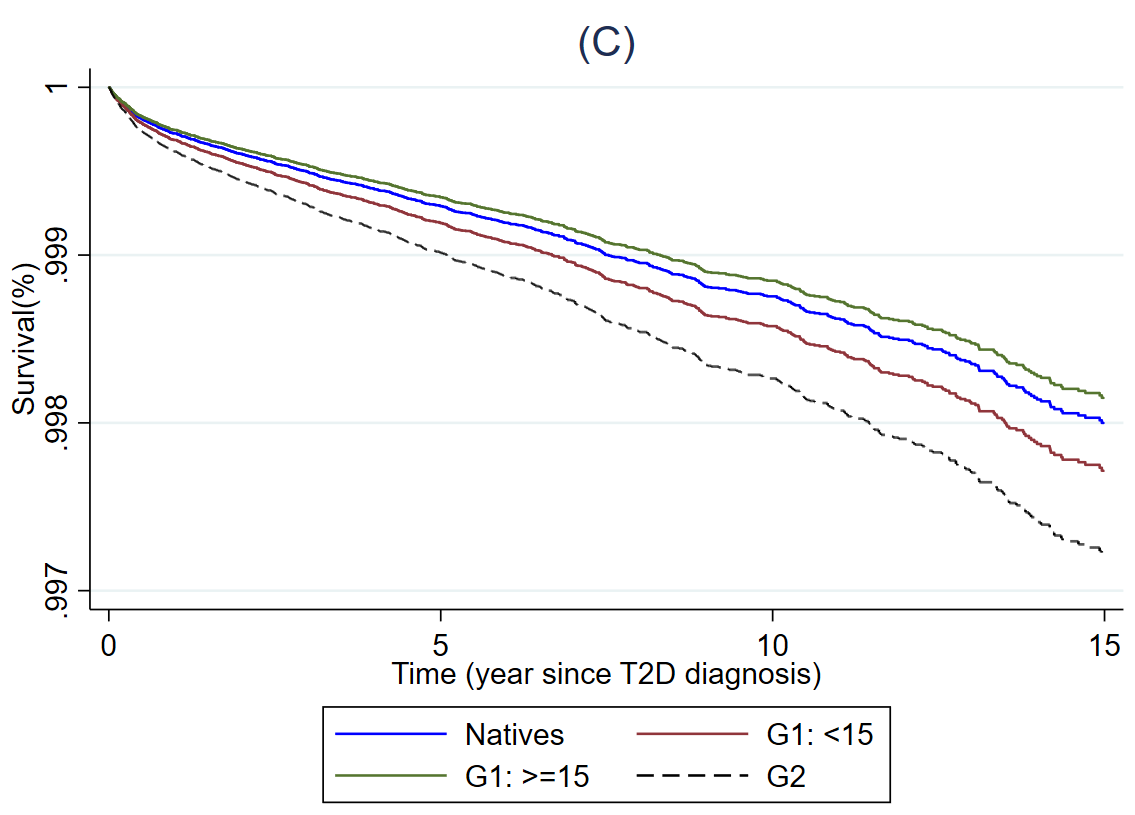 |
| **Bladder** | | | |
| 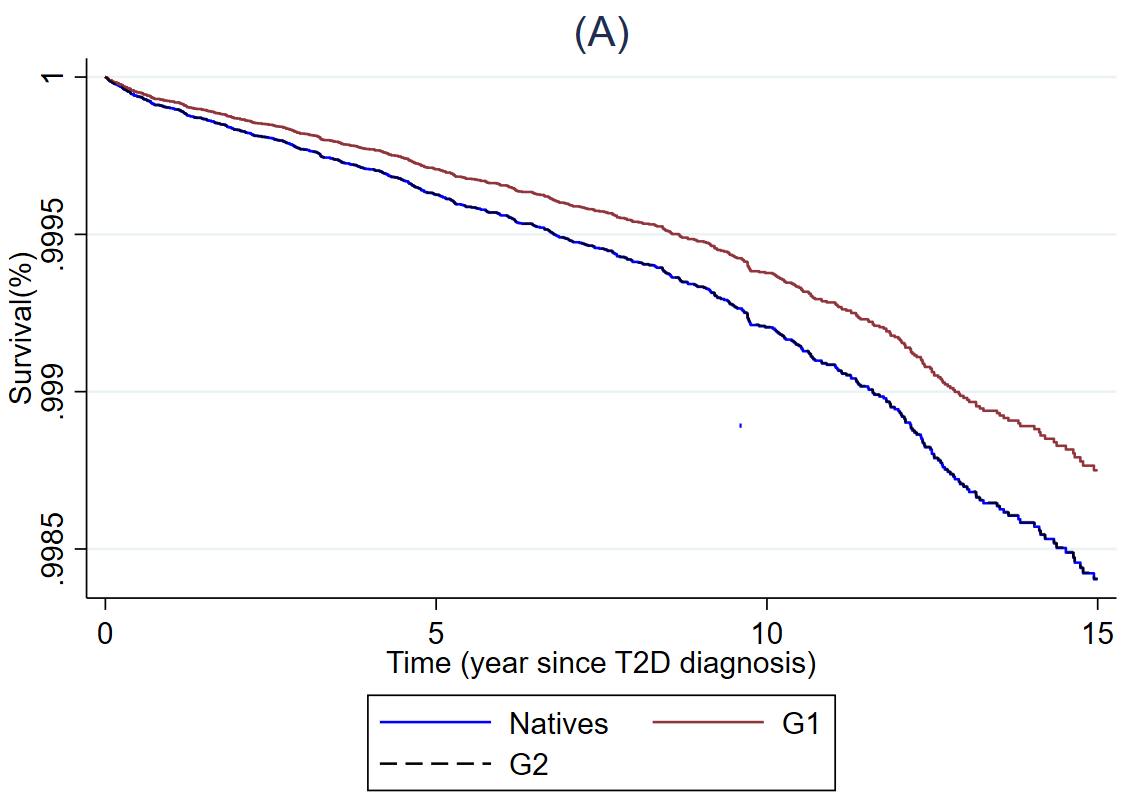 | | 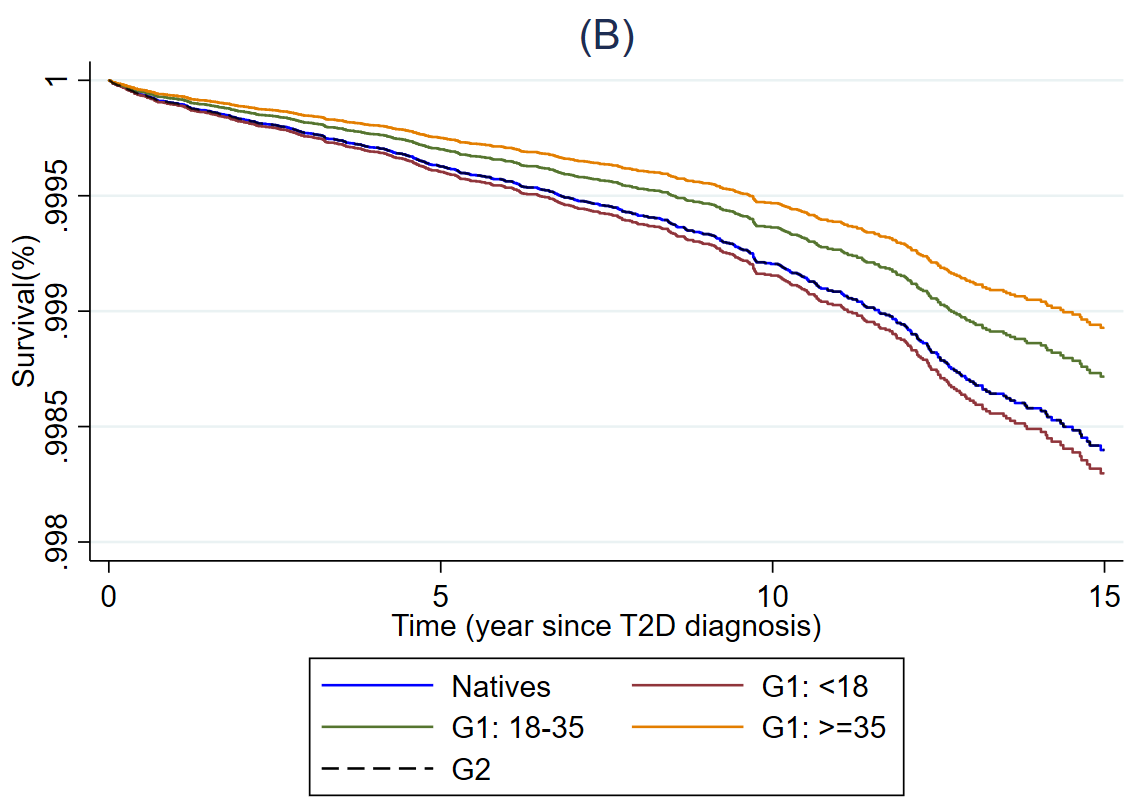 | 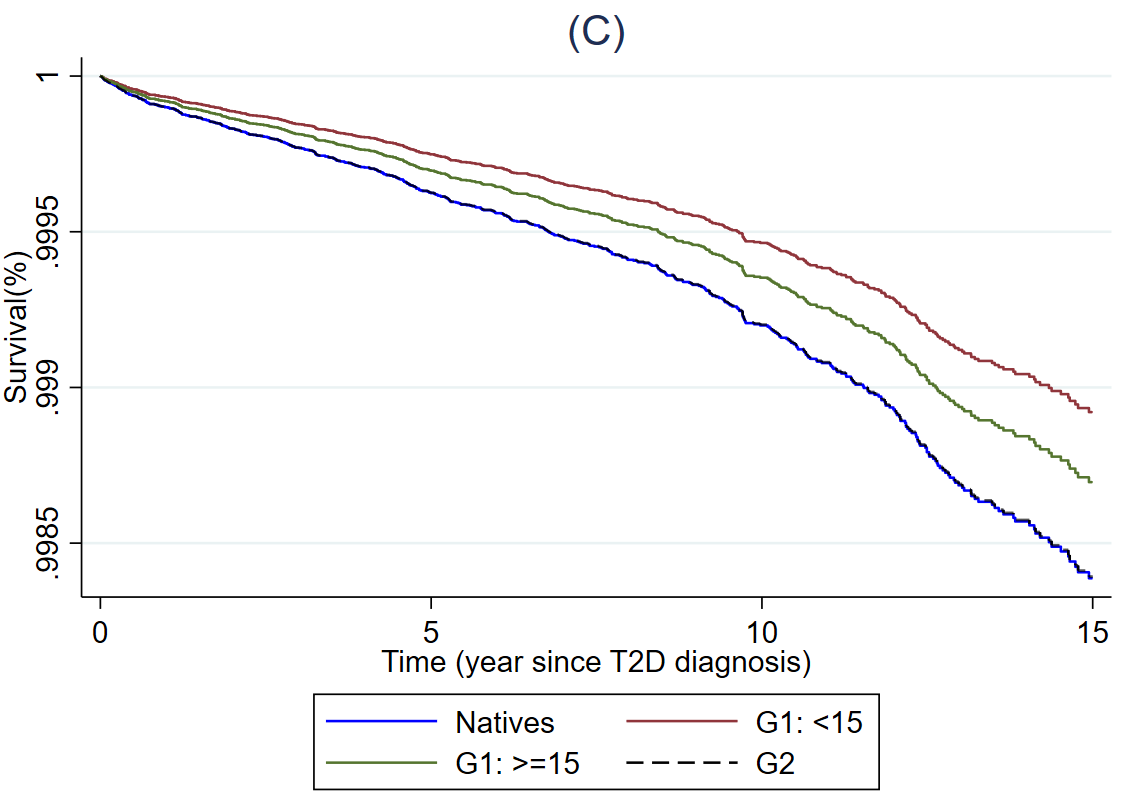 |
| **Breast** | | | |
|  | | 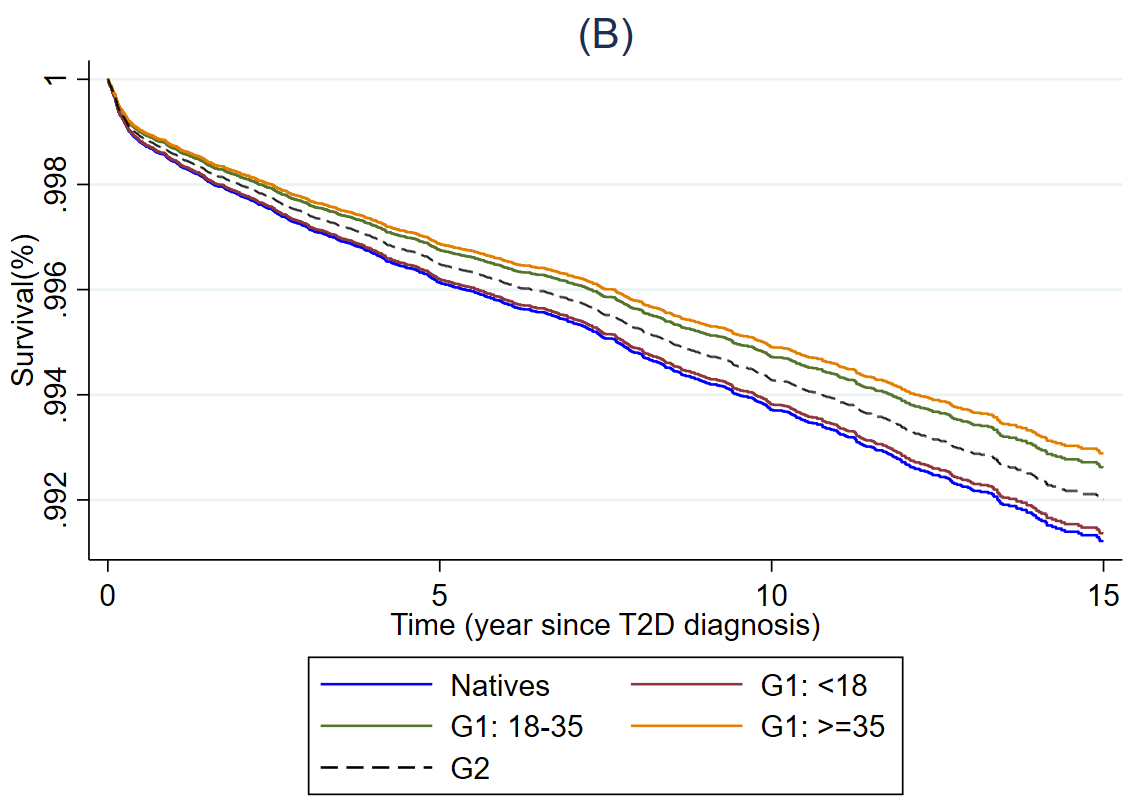 | 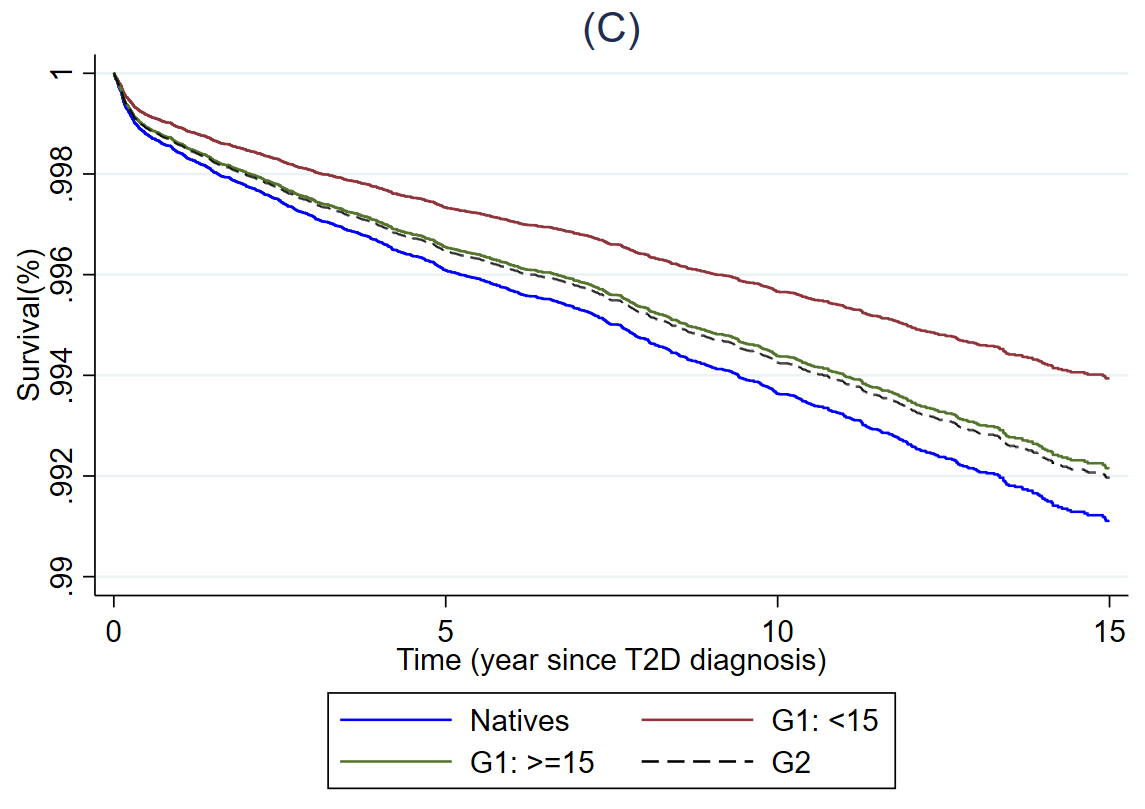 |
| **Endometrial** | | | |
| 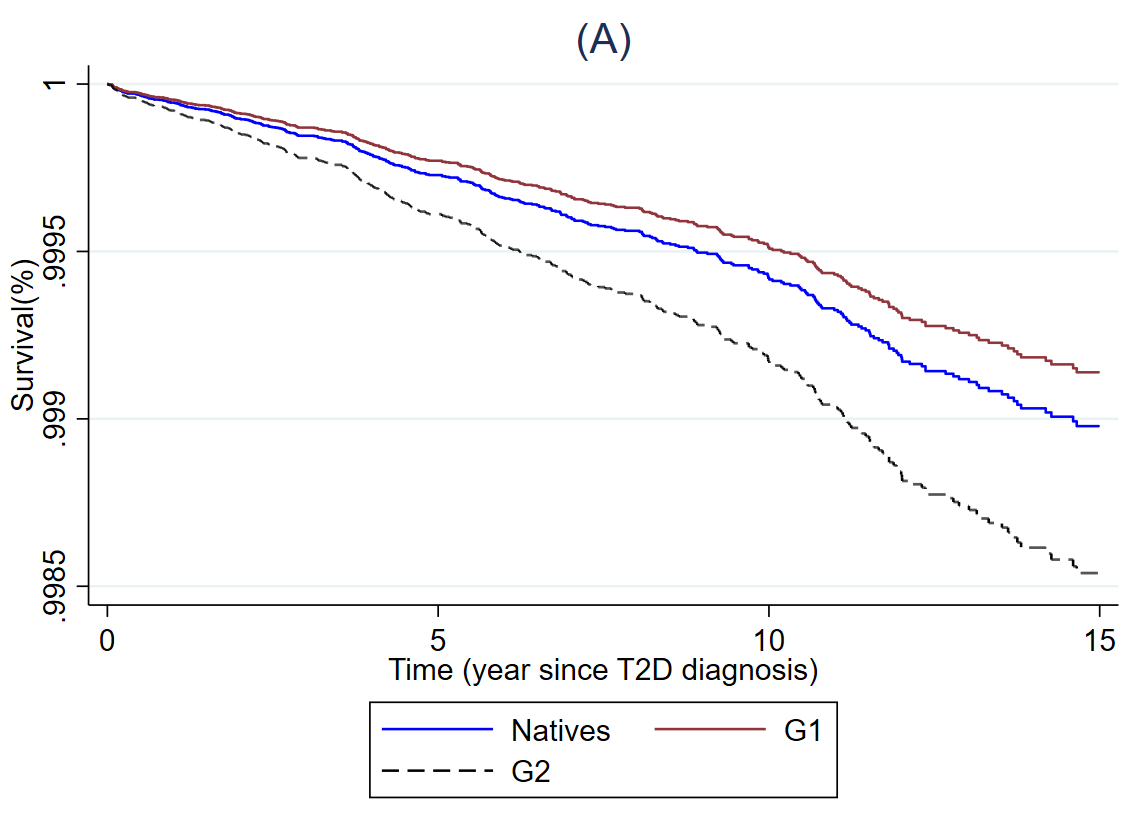 | | 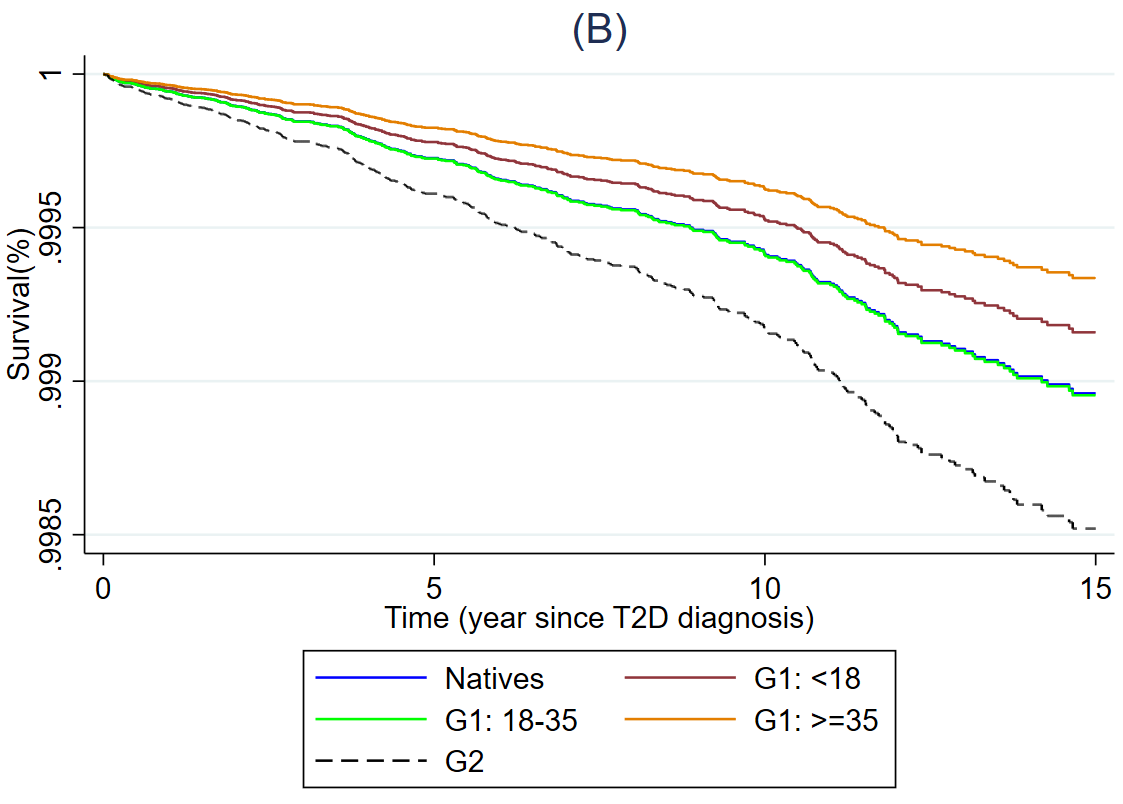 | 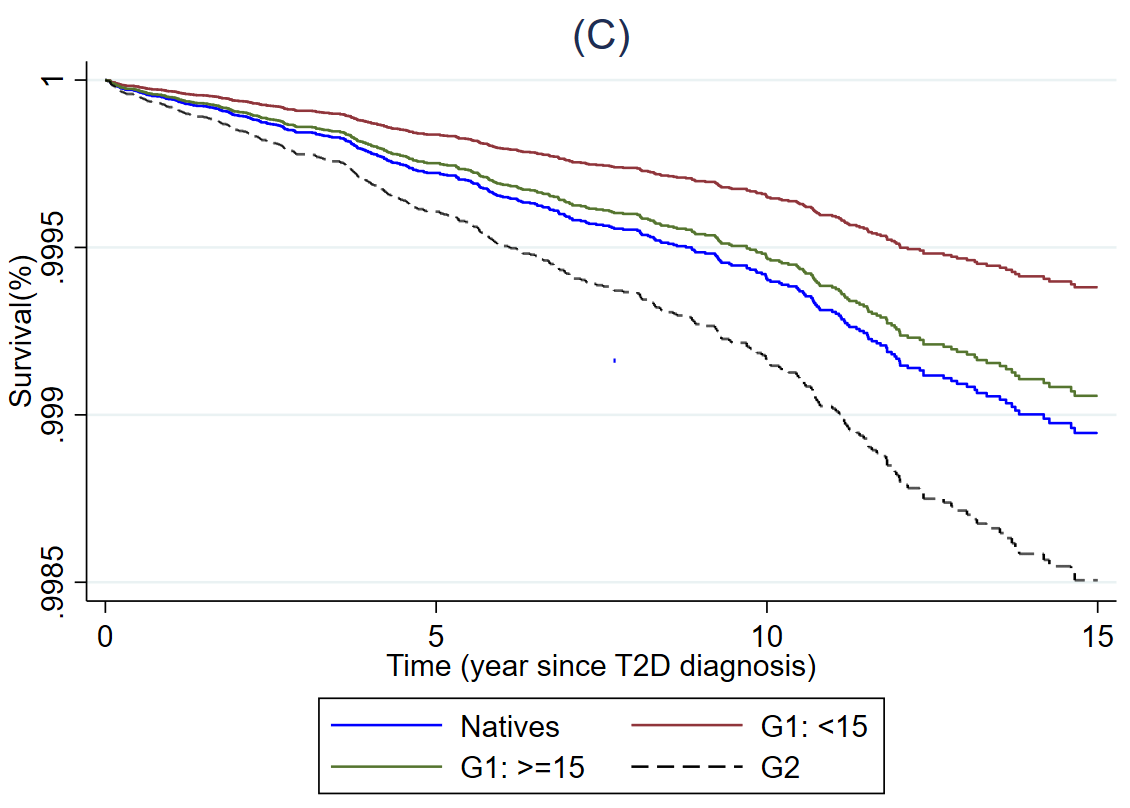 |

**sFigure 1:** Survival curve probability for **all-site and site-specific cancer mortality** in type 2 diabetes patients by immigrant generation groups compared to natives. Survival curves adjusted for sex, age, income quantiles, educations, marital status, employment, comorbidities, and calendar period. (A): Natives, G1, and G2; (B): Natives, G1 (by age (in years) at arrival), and G2; (C) - Natives, G1 (by year in Sweden at type 2 diabetes diagnosis), and G2

Total T2D cases diagnosed between 2006 and 2021, identified from **NPR ^a^ and PDR** ^b^

(**n=630, 671**)

**Excluded:**

Diagnosis with T2D before age 35 (n=44,052)

Missing data for birth data/age (n=10, 144)

Immigrants who died within two years post-diagnosis for (n=170)

Merge with total population registry, and cause of death registry, and multi-generation registry, using unique personal identification number

Merge with LISA registry ^D^

**(**Before exclusion**:** n=532, 973**)**

**Final study population**

(**n= 478,607, aged above or equal 35)**

Natives = 313,899

G1 = 135,787

G2 = 28,921

**Excluded:**

Immigrant’s origin and generation groups not defined (n=98,314), and untestable information on age at arrival (n=104)

**sFigure 2 – Final study population and exclusion criteria, Sweden, 2006-2021**

^a^NPR (National patient registry) – T2D cases were identified from this registry using International Classification of Diseases (*ICD-10*: E10-E15)

^b^PDR (Prescribed Drug registry) – T2D cases were identified from this registry using Anatomical Therapeutic Chemical (ATC) classification

code starting with “A10”

^C^LISA (The Longitudinal Database of Health, Insurance, and Labor Market Studies) - includes multiple observations of study participants’ socio

economic characteristics data.

Abbreviations: T2D – Type 2 diabetes; G1 – First-generation immigrants; G2 – Second-generation immigrants

**sTable 2 (a)**: Age and sex standardized cancer mortality ratio (SMR_atio_) - calculated by dividing the ratio of standardized cancer mortality rate in immigrants with vs. without type 2 diabetes to the ratio among natives with vs. without type 2 diabetes, both sexes, 2006 to 2021

| **Immigrant groups** | **Type 2 diabetes** | **Person years** | **All cancer** | | | **Liver** | | | **Kidney** | | | **Colorectal** | | |
| --- | --- | --- | --- | --- | --- | --- | --- | --- | --- | --- | --- | --- | --- | --- |
|  |  |  | **Cases** | **SMRate*** | **SMRatio (95% CI)** | **Cases** | **SMRate*** | **SMRatio (95% CI)** | **Cases** | **SMRate*** | **SMRatio (95% CI)** | **Cases** | **SMRate*** | **SMRatio (95% CI)** |
| Natives | With | 2,503,034 | 21,401 | 578.2 | Ref | 947 | 22.8 | Ref | 628 | 14.8 | Ref | 2,153 | 56.9 | Ref |
|  | Without | 54,688,331 | 139,619 | 311.8 |  | 3,700 | 8.1 |  | 3,552 | 8.1 |  | 17,372 | 38.9 |  |
| G1 | With | 1,048,753 | 6,074 | 464.0 | **0.74 (0.72, 0.76**) | 307 | 21.6 | **0.70 (0.61, 0.81)** | 189 | 14.3 | 1.02 (0.85, 1.22) | 545 | 42.3 | **0.75 (0.67, 0.83)** |
|  | Without | 14,431,273 | 34,586 | 337.1 |  | 1,114 | 10.9 |  | 838 | 7.7 |  | 3,984 | 38.6 |  |
| **By age at arrival** | | | | | | | | | | | | | | |
| G1: age <18  at arrival | With | 129,928 | 795 | 572.4 | **0.90 (0.83, 0.97)** | 41 | 26.7 | 0.97 (0.68, 1.38) | 23 | 15.8 | 0.99 (0.62, 1.58) | 84 | 62.3 | 1.06 (0.84, 1.35) |
|  | Without | 2,492,129 | 4,272 | 344.8 |  | 136 | 9.8 |  | 91 | 8.7 |  | 490 | 40.0 |  |
| G1: age 18-  35 at arrival | With | 497,766 | 3,060 | 495.5 | **0.76 (0.73, 0.79)** | 141 | 20.4 | **0.66 (0.55, 0.81)** | 95 | 15.4 | 0.92 (0.72, 1.16) | 282 | 45.7 | **0.78 (0.68, 0.89)** |
|  | Without | 7,611,395 | 18,394 | 351.9 |  | 564 | 10.9 |  | 471 | 9.2 |  | 2,090 | 39.9 |  |
| G1: age >= 35  at arrival | With | 402,937 | 1,878 | 375.9 | **0.69 (0.65, 0.73)** | 110 | 20.3 | **0.64 (0.51, 0.80)** | 64 | 11.9 | **1.05** (0.78, 1.41) | 150 | 30.7 | **0.64 (0.54, 0.76)** |
|  | Without | 4,053,376 | 9,168 | 293.5 |  | 355 | 11.3 |  | 208 | 6.9 |  | 1,031 | 32.8 |  |
| **By country of origin** | | | | | | | | | | | | | | |
| G1: Nordic | With | 208,803 | 2,215 | 639.2 | 0.89 (0.78, 1.01) | 100 | 25.3 | **0.76 (0.60, 0.95)** | 75 | 23.1 | **1.23** (0.94, 1.59) | 200 | 54.5 | 0.87 (0.75, 1.01) |
|  | Without | 3,248,703 | 16,165 | 388.2 |  | 469 | 11.9 |  | 419 | 10.3 |  | 1,851 | 42.9 |  |
| G1: Western | With | 267,264 | 2,166 | 550.0 | **0.88 (0.84, 0.92)** | 93 | 21.4 | 0.89 (0.70, 1.14) | 74 | 19.2 | **1.25** (0.98, 1.61) | 206 | 49.7 | **0.85 (0.73, 0.99)** |
|  | Without | 4,612,180 | 12,404 | 337.9 |  | 303 | 8.5 |  | 300 | 8.4 |  | 1,485 | 40.1 |  |
| G1: Non-Western | With | 565,941 | 1,693 | 325.9 | **0.74 (0.70, 0.79)** | 115 | 21.1 | **0.61 (0.49, 0.76)** | 42 | 7.8 | 0.88 (0.62, 1.28) | 145 | 27.9 | **0.75 (0.62, 0.90)** |
|  | Without | 6,570,390 | 6,017 | 236.4 |  | 342 | 12.2 |  | 119 | 4.8 |  | 648 | 25.5 |  |
| G2: All | With | 222,627 | 1,232 | 522.0 | 0.93 (0.86, 1.00) | 68 | 21.6 | 0.86 (0.65, 1.13) | 51 | 18.2 | **1.30** (0.95, 1.76) | 124 | 49.6 | 0.97 (0.80, 1.17) |
|  | Without | 5,781,599 | 7,939 | 303.0 |  | 273 | 8.9 |  | 169 | 7.7 |  | 926 | 35.0 |  |
| G2: Nordic | With | 140,224 | 805 | 524.3 | 0.92 (0.83, 1.01.) | - | - | - | - | - | - | - | - | - |
|  | Without | 3,462,259 | 5,207 | 307.9 |  | - | - | - | - | - | - | - | - | - |
| G2: Western | With | 65,976 | 379 | 504.1 | 0.99 (0.88, 1.10) | - | - | - | - | - | - | - | - | - |
|  | Without | 1,972,918 | 2,380 | 274.9 |  | - | - | - | - | - | - | - | - | - |
| G2: Non-Western | With | 5,595 | 18 | 428.1 | 0.87 (0.53, 1.42) | - | - | - | - | - | - | - | - | - |
|  | Without | 326,608 | 122 | 265.7 |  | - | - | - | - | - | - | - | - | - |

**sTable 2 (a) - continued**

| **Immigrant groups** | **Type 2 diabetes** | **Person years** | **Pancreas** | | | **Bladder** | | | **Esophageal** | | |
| --- | --- | --- | --- | --- | --- | --- | --- | --- | --- | --- | --- |
|  |  |  | **Cases** | **SMRate*** | **SMRatio (95% CI)** | **Cases** | **SMRate*** | **SMRatio (95% CI)** | **Cases** | **SMRate*** | **SMRatio (95% CI)** |
| Natives | With | 2,503,034 | 4,040 | 104.9 | Ref | 484 | 10.9 | Ref | 403 | 9.4 | Ref |
|  | Without | 54,688,331 | 9,962 | 21.5 |  | 3,490 | 9.2 |  | 3,573 | 7.6 |  |
| G1 | With | 1,048,753 | 979 | 74.3 | **0.73 (0.67, 0.79)** | 121 | 9.1 | **0.75 (0.61, 0.93)** | 97 | 7.2 | 0.84 (0.67, 1.07) |
|  | Without | 14,431,273 | 2,194 | 20.9 |  | 898 | 10.2 |  | 684 | 6.9 |  |
| G1: age <18  at arrival | With | 129,928 | 153 | 107.2 | 0.94 (0.77, 1.14) | 16 | 14.0 | **1.36** (0.79, 2.34) | 13 | 9.1 | 0.94 (0.52, 1.69) |
|  | Without | 2,492,129 | 295 | 23.4 |  | 80 | 8.7 |  | 102 | 7.8 |  |
| G1: age 18-  35 at arrival | With | 497,766 | 528 | 83.8 | **0.75 (0.68, 0.84)** | 62 | 9.8 | 0.75 (0.57, 1.00) | 51 | 7.8 | 0.91 (0.67, 1.25) |
|  | Without | 7,611,395 | 1,231 | 22.8 |  | 478 | 11.0 |  | 345 | 6.9 |  |
| G1: age >=35  at arrival | With | 402,937 | 249 | 52.4 | **0.66 (0.57, 0.77)** | 38 | 7.5 | 0.72 (0.50, 1.03) | 29 | 5.7 | 0.79 (0.53, 1.19) |
|  | Without | 4,053,376 | 514 | 16.2 |  | 247 | 8.8 |  | 175 | 5.8 |  |
| G1: Nordic | With | 208,803 | 406 | 106.5 | **0.87 (0.77, 0.98)** | 43 | 10.9 | 0.84 (0.61, 1.17) | 43 | 12.7 | 1.06 (0.76, 1.47) |
|  | Without | 3,248,703 | 1,097 | 25.0 |  | 403 | 10.9 |  | 371 | 9.7 |  |
| G1: Western | With | 267,264 | 352 | 91.4 | 0.90 (0.79, 1.03) | 51 | 11.3 | 0.82 (0.61, 1.12) | 24 | 5.7 | 0.78 (0.51, 1.21) |
|  | Without | 4,612,180 | 774 | 20.7 |  | 393 | 11.6 |  | 207 | 5.9 |  |
| G1: Non-Western | With | 565,941 | 229 | 41.2 | **0.62 (0.52, 0.74)** | 28 | 7.1 | **1.02** (0.66, 1.56) | 30 | 6.0 | **1.16** (0.76, 1.75) |
|  | Without | 6,570,390 | 323 | 13.6 |  | 102 | 5.9 |  | 106 | 4.2 |  |
| G2 | With | 222,627 | 223 | 85.8 | 0.90 (0.76, 1.05) | 23 | 11.4 | **1.07** (0.68, 1.67) | 21 | 9.6 | 1.00 (0.63, 1.58) |
|  | Without | 5,781,599 | 542 | 19.6 |  | 180 | 9.0 |  | 210 | 7.8 |  |

* Age standardized using European Standard Population (ESP) and expressed per 100,000 person years. Abbreviations - G1 (First generation immigrants), G2 (second generation immigrants)

A log-transformation approach was used for calculating confidence intervals for the standardized mortality ratio (SMR). Variances were estimated via the Delta Method, and 95% confidence intervals were derived by exponentiating the log-based limits.

**sTable 2 (b)**: Age standardized cancer mortality ratio (SMRatio) - calculated by dividing the ratio of standardized cancer mortality rate in immigrants with vs. without type 2 diabetes to the ratio among natives with vs. without type 2 diabetes, females, 2006 to 2021

| **Immigrant groups** | **Type 2 diabetes** | **Person years** | **Breast** | | | **Endometrial** | | |
| --- | --- | --- | --- | --- | --- | --- | --- | --- |
|  |  |  | **Cases** | **SMRate*** | **SMRatio (95% CI)** | **Cases** | **SMRate*** | **SMRatio (95% CI)** |
| Natives | With | 1,005,999 | 959 | 75.5 | Ref | 275 | 17.0 | Ref |
|  | Without | 27,858,374 | 9,979 | 38.7 |  | 2,119 | 8.7 |  |
| G1 | With | 480,343 | 338 | 59.3 | **0.79 (0.69, 0.91)** | 94 | 14.5 | 0.81 (0.63, 1.04) |
|  | Without | 7,664,851 | 2,526 | 38.3 |  | 571 | 9.2 |  |
| G1: age <18  at arrival | With | 58,883 | 45 | 72.5 | 0.91 (0.66, 1.24) | 8 | 10.6 | 0.59 (0.28, 1.24) |
|  | Without | 1,285,530 | 364 | 40.9 |  | 66 | 9.2 |  |
| G1: age 18-  35 at arrival | With | 224,605 | 156 | 61.0 | **0.79 (0.66, 0.94)** | 50 | 16.9 | 0.89 (0.64, 1.23) |
|  | Without | 4,131,621 | 1,337 | 39.5 |  | 310 | 9.7 |  |
| G1: age >=35  at arrival | With | 187,415 | 121 | 48.7 | **0.74 (0.61, 0.91)** | 33 | 12.6 | 0.78 (0.53, 1.17) |
|  | Without | 2,077,214 | 656 | 33.6 |  | 149 | 8.2 |  |
| G1: Nordic | With | 100,853 | 112 | 108.6 | **1.35 (1.10, 1.66)** | 33 | 15.4 | 0.95 (0.65, 1.39) |
|  | Without | 1,909,528 | 1,078 | 41.1 |  | 234 | 8.3 |  |
| G1: Western | With | 124,003 | 113 | 69.1 | 0.89 (0.72, 1.09) | 34 | 16.7 | 0.77 (0.53, 1.13) |
|  | Without | 2,423,645 | 881 | 39.9 |  | 237 | 11.1 |  |
| G1: Non-Western | With | 255,487 | 113 | 44.0 | **0.78 (0.63, 0.96)** | 27 | 10.0 | **0.63 (0.41, 0.98)** |
|  | Without | 3,331,678 | 567 | 29.0 |  | 100 | 8.1 |  |
| G2 | With | 87,455 | 60 | 67.5 | 0.97 (0.74, 1.27) | 23 | 21.9 | **1.29** (0.81, 2.04) |
|  | Without | 2,888,896 | 694 | 35.6 |  | 119 | 8.7 |  |

* Age standardized using the 2013 European Standard Population (ESP) and expressed per 100,000 person years. Abbreviations - G1 (First generation immigrants), G2 (second generation immigrants)

sTable 3: Sensitivity analysis (excluding individuals diagnosed with cancer before type 2 diabetes) for examining all-site cancers and site-specific cancer mortality in type 2 diabetes patients by generation groups: G1 (stratified further by country of origin, age at arrival, and time in Sweden at the time of type 2 diabetes of diagnosis) and G2, compared to natives, Sweden, 2006-2021.

| **Cancer type** | **G1: HR (95% CI)** | | | | | | **G2** |
| --- | --- | --- | --- | --- | --- | --- | --- |
|  | **All** | **<18 at arrival** | **18-35 at arrival** | **>= 35 at arrival** | **Time in Sweden: <15 years** | **Time in Sweden: >=15 years** | **All** |
| All site | **0.87 (0.84, 0.91)** | **1.15 (1.06, 1.25)** | **0.91 (0.86, 0.95)** | **0.69 (0.65, 0.74)** | **0.65 (0.60, 0.72)** | **0.90 (0.86, 0.93)** | 1.03 (0.96, 1.11) |
| Colorectal | **0.78 (0.69, 0.89)** | 1.19 (0.92, 1.56) | **0.83 (0.71, 0.97)** | **0.52 (0.42, 0.66)** | **0.44 (0.32, 0.61)** | **0.83 (0.73, 0.94)** | 0.98 (0.78, 1.22) |
| Liver | 0.91 (0.78, 1.06) | 1.16 (0.83, 1.62) | 0.91 (0.75, 1.10) | **0.77 (0.59, 0.99)** | **0.60 (0.42, 0.85)** | 0.95 (0.81, 1.11) | 1.10 (0.83, 1.44) |
| Pancreas | **0.81 (0.75, 0.89)** | **1.24 (1.03, 1.49)** | **0.88 (0.79, 0.98)** | **0.49 (0.42, 0.59)** | **0.45 (0.36, 0.58)** | **0.85 (0.78, 0.94)** | 0.94 (0.79, 1.10) |
| Kidney | 0.86 (0.68, 1.07) | 1.13 (0.67, 1.89) | 0.82 (0.61, 1.09) | 0.84 (0.59, 1.20) | 0.99 (0.62, 1.59) | 0.84 (0.66, 1.07) | **1.47 (1.03, 2.11)** |
| Bladder | **0.73 (0.57, 0.94)** | 1.11 (0.63, 1.98) | 0.81 (0.59, 1.10) | **0.43 (0.26, 0.70)** | 0.68 (0.36, 1.29) | **0.72 (0.55, 0.94)** | 0.93 (0.56, 1.55) |
| Breast: females | 0.96 (0.77, 1.21) | 1.00 (0.58, 1.72) | 0.95 (0.71, 1.26) | 0.85 (0.59, 1.22) | 0.91 (0.58, 1.43) | 0.92 (0.72, 1.18) | 1.17 (0.75, 1.81) |
| Endometrial | 0.84 (0.61, 1.16) | NR | 1.06 (0.73, 1.56) | 0.68 (0.40, 1.16) | 0.58 (0.27, 1.22) | 0.88 (0.63, 1.24) | **1.74 (1.07, 2.85)** |

| **Cancer type** | **G1: HR (95% CI)** | | | **G2: HR (95% CI)** | | |
| --- | --- | --- | --- | --- | --- | --- |
|  | **Nordic** | **Western exc. Nordic** | **Non- Western** | **Nordic** | **Western exc. Nordic** | **Non-western^‡^** |
| All site | **1.06 (1.00, 1.12)** | 0.95 (0.90, 1.00) | **0.59 (0.55, 0.63**) | 1.00 (0.92, 1.09) | 1.03 (0.90, 1.16) | 0.93 (0.50, 1.74) |
| Colorectal | 1.01 (0.85, 1.20) | 0.90 (0.76, 1.08) | **0.48 (0.39, 0.60)** | 0.81 (0.60, 1.09) | 1.17 (0.82, 1.70) | NR |
| Liver | 1.08 (0.87, 1.35) | 0.92 (0.73, 1.17) | **0.77 (0.61, 0.97)** | 1.05 (0.75, 1.46) | 1.03 (0.62, 1.69) | NR |
| Pancreas | **1.15 (1.02, 1.30)** | **0.87 (0.76, 0.99)** | **0.46 (0.39, 0.54)** | 1.01 (0.83, 1.22) | 0.78 (0.56, 1.07) | NR |
| Kidney | 1.03 (0.75, 1.43) | 1.08 (0.78, 1.47) | **0.52 (0.35, 0.77)** | 1.33 (0.84, 2.10) | **1.84 (1.03, 3.30)** | NR |
| Bladder | 0.92 (0.64, 1.31) | 0.81 (0.56, 1.16) | **0.45 (0.29, 0.73)** | 0.62 (0.29, 1.31) | 1.43 (0.68, 3.04) | NR |
| Breast: females | 1.18 (0.87, 1.62) | 0.94 (0.67, 1.32) | 0.77 (0.54, 1.11) | 0.94 (0.52, 1.67) | 1.73 (0.88, 3.38) | NR |
| Endometrial | 0.97 (0.62, 1.53) | 0.92 (0.57, 1.47) | **0.57 (0.33, 0.99)** | **2.23 (1.32, 3.75)** | NR | NR |

Significant results (p<0.05) highlighted in bold**.**

Nordic (Finland, Denmark, Norway, Iceland), Western excl. Nordic (all Europe excl. Nordic, USA, Canada, and Oceanian i.e., Australia and New Zealand), and non-Western (Latin America, Asia, Africa and Middle East). NR: the number of cases were too small (n < 5), and thus HRs are not reported.

|  |  |
| --- | --- |
| (a) | (b) |

sFigure (3) – Sensitivity analysis (excluding individuals diagnosed with cancer prior to type 2 diabetes): adjusted hazard ratios for all-site combined mortality among type 2 diabetes patients aged 35 and above, comparing **(a)** G1 to Natives and **(b)** G2 to Natives, as a function of year of type 2 diabetes diagnosis between 2006 and 2021, Sweden. Variables used for adjustment were age, calendar period, SES (income, education, employment status), marital status, and selected comorbidities (CVDs, obesity, and other disorders including pulmonary, renal or kidney, viral hepatitis, liver, depression and anxiety, and dementia).
